# Lectin-Micropatterned Biointerfaces Regulate Cytoskeletal Organization and Viscoelastic Phenotypes of Bladder Cancer Cells

**DOI:** 10.1021/acsomega.6c04675

**Published:** 2026-08-06

**Authors:** Joanna Zemła, Justyna Śmiałek-Bartyzel, Bartosz Brzuchacz, Małgorzata Lekka

**Affiliations:** † Institute of Nuclear Physics, Polish Academy of Sciences, Krakow 31342, Poland; ‡ Faculty of Chemical Engineering and Technology, Cracow University of Technology, Warszawska 24, Kraków 30155, Poland

## Abstract

Altered glycosylation is a hallmark of cancer progression and provides opportunities to modulate cell adhesion through glycan–lectin interactions. In this study, we investigate how lectin-mediated adhesion and substrate micropatterns influence cytoskeletal organization and viscoelastic properties of bladder cancer cells. Bladder cancer cell lines—HCV29, 5637, and T24 cells—representing different malignancy stages were cultured on homogeneous and micropatterned lectin-functionalized substrates. Lectins recognizing N-glycans (*Dolichos biflorus agglutinin*, DBA; *Wheat germ agglutinin*, WGA) and fucosylated glycans (*Lotus tetragonolobus lectin*, LTL) were used to selectively position bladder cancer cells via glycan-dependent adhesion. Cell morphology and cytoskeletal organization were analyzed using fluorescence microscopy, while nanomechanical and rheological properties were quantified by atomic force microscopy (AFM) using nanoindentation and stress-relaxation experiments. All studied lectins showed affinity for HCV29, 5637, and T24 cells. HCV29 and T24 cells displayed favorable interactions with N-glycan-binding lectins, whereas 5637 cells showed reduced sensitivity to lectin chemistry. Lectin-striped micropatterns induced pronounced morphological changes, including cell elongation and cytoskeletal polarization. AFM measurements demonstrated that both surface chemistry and surface confinement modulate viscoelastic phenotype of cells, with an increase in apparent Young’s modulus observed for cells growing on lectin stripes. Viscoelastic parameters such as the equilibrium elastic modulus and Deborah number increased for 5637 and T24 cells, indicating a shift toward more solid-like behavior. We found that lectin affinity alone did not determine cell positioning or mechanical adaptation. Instead, cell-line–specific responses emerged from the combined effects of initial adhesion cues, geometric confinement, and subsequent adaptive cellular processes. Our findings show that lectin-based micropatterning provides a biologically relevant modulating cell organization and probing the mechanobiological behavior of bladder cancer cells, promising a framework for developing automated biomechanical assays and mechanophenotypic biomarkers relevant to cancer research.

## Graphical abstract

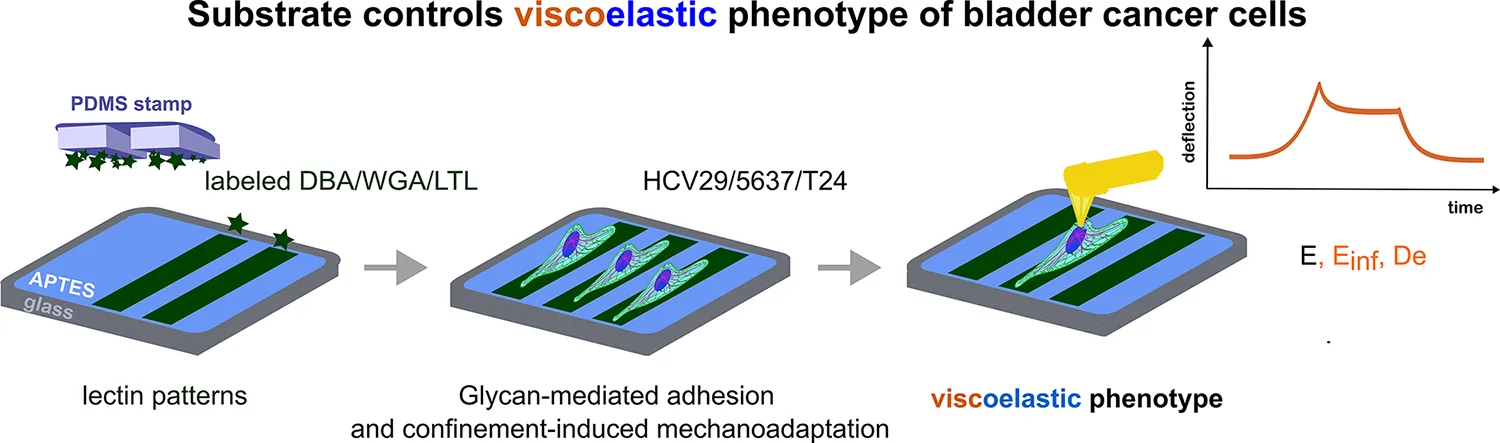

## 1 Introduction

Cell-surface glycans form a dynamic molecular interface that regulates cell adhesion, signaling, and mechanical interactions with the extracellular environment.^1–3^ In cancer, the cellular glycome undergoes extensive remodeling, leading to altered glycan structures on membrane proteins and lipids that influence cell–cell and cell–matrix interactions.^1–5^ In bladder cancer, significant changes have been reported in N-glycan structures and their terminal modifications, including variations in fucosylation and sialylation patterns.^4^ Previous studies have shown that low-grade bladder cancer cells frequently express highly fucosylated N-glycans and Lewis-type epitopes such as Lewis X. In contrast, high-grade tumor cells often display reduced expression of these structures and distinct glycosylation signatures.^6^ Such glycan alterations affect the organization and activity of adhesion receptors such as integrins and cadherins, thereby modulating cell adhesion, migration, and signaling pathways associated with tumor progression.^4,6–8^

Lectins, carbohydrate-binding proteins with high specificity toward defined glycan motifs, provide a versatile strategy for probing cell-surface glycosylation and engineering glycan-responsive biointerfaces.^9,10^ By selectively recognizing glycan structures such as N-glycans or fucosylated epitopes, lectins can mediate glycan-dependent cell adhesion and enable the development of functionalized surfaces that interact with cells through their glycome.^11^ Lectin-functionalized materials have therefore emerged as promising tools for studying glycan-mediated cell interactions and for capturing or organizing cells based on their glycosylation profiles.^11,12^ When combined with techniques such as microcontact printing (μCP),^10,13^ such substrates can impose defined spatial constraints on adherent cells, thereby controlling cell geometry, cytoskeletal organization, and adhesion distribution.^14^ Beyond fundamental studies of cell–surface interactions, they may also facilitate controlled cell positioning for high-throughput mechanobiological measurements or diagnostic platforms (biosensors) that exploit differences in cellular glycosylation.

Cell geometry and cytoskeletal architecture are well-known to regulate cellular mechanical behavior. Spatial confinement of adherent cells can induce cytoskeletal polarization, alter actin organization, and modulate intracellular tension, ultimately affecting cell stiffness and viscoelastic properties.^14^ Such mechanical characteristics have been increasingly recognized as mechanophenotypic descriptors that reflect cellular state and disease progression. Atomic force microscopy (AFM) provides a powerful approach for quantifying cellular nanomechanics, enabling measurements of both elastic and time-dependent viscoelastic properties at the single-cell level.^15–17^

Despite growing interest in glycan-mediated cell adhesion, most studies of cell-related micropatterning rely on extracellular matrix proteins.^14,18–20^ Comparatively little is known about how glycan-specific adhesion cues, combined with surface confinement, influence the mechanical phenotype of cancer cells. Recently, we demonstrated that the nanomechanical properties of immortalized urothelial control HCV29 cells are insensitive to lectin coating. In contrast, for noninvasive and invasive HT1376 and T24 cancer cells, we observed changes in the apparent Young’s modulus when cultured on homogeneous lectin-functionalized substrates.^21^

In this work, we investigate how lectin-mediated adhesion cues and geometric confinement on patterned substrates modulate the viscoelasticity of bladder cancer cells. Bladder cancer cells (i.e., immortalized urothelial control HCV29 and cancerous 5637 and T24 cells, originating from different stages of malignancy) were cultured on homogeneous lectin-functionalized substrates and on lectin–APTES micropatterned surfaces fabricated by microcontact printing. Lectins recognizing N-glycans, i.e., *Dolichos biflorus agglutinin* (DBA) and *Wheat germ agglutinin* (WGA), and fucosylated glycans (*Lotus tetragonolobus lectin*, LTL) were used to functionalize substrates, on which cells were grown. Cytoskeletal organization was analyzed using fluorescence microscopy, while cellular nanomechanics and rheological behavior were quantified using AFM working in nanoindentation and stress-relaxation modes.

We found that geometric confinement on lectin-patterned substrates, together with lectin-mediated adhesion cues, modulates the mechanical behavior and fluidity of the bladder cancer cells. Fluorescence imaging revealed increased actin bundle formation in nonmalignant HCV29 and cancerous T24 cells, whereas 5637 cells exhibited a more elongated morphology with MTs aligned along the lectin patterns. Our results demonstrate that a high lectin affinity alone does not determine cell positioning or mechanical adaptation. Instead, the observed viscoelastic phenotype arises from cell-specific responses to the combined effects of initial adhesion cues, geometric confinement, and subsequent adaptive cellular processes.

## 2 Materials and Methods

### 2.1 Cells

The following bladder cell lines of epithelial origin were used in the study, namely, nontumorigenic immortalized urothelial cell line (HCV29 cells, Institute of Experimental Therapy, Wroclaw, Poland), urinary bladder cell carcinoma (5637 cells, grade II, ATCC, LGC Standards), and transitional cell carcinoma (T24 cells, ATCC, LGC Standards). HCV29 and T24 cells were cultured in RPMI-1640 medium (Merck, Poznań, Poland) supplemented with 10% fetal bovine serum (FBS, Sigma-Aldrich, Poznań, Poland), and 5637 cells were cultured in RPMI-1640 medium with 10% FBS and 1% HEPES (4-(2-hydroxyethyl)­piperazine-1-ethanesulfonic acid, Merck, Poznań, Poland) and 1% sodium pyruvate (Merck, Poznań, Poland). Plastic culture flasks (TPP, 25 cm^2^, Genos, Łódź, Poland) were used for cell cultures carried out in a CO_2_ incubator (Nuaire, USA) at 37 °C in a 95% air/5% CO_2_ atmosphere.

### 2.2 Lectins

Based on our previous research,^12,13,22^ the following lectins were selected for the study. They were *Wheat germ agglutinin* (WGA), *Dolichos biflorus agglutinin* (DBA), and *Lotus tetragonolobus lectin* (LTL). In parallel, fluorescently labeled lectins were also used. They were rhodamine-labeled WGA (WGA-TRITC, excitation/emission 550/575 nm), and fluorescein-labeled DBA and LTL lectins (DBA-FITC and LTL-FITC, excitation/emission 495/515 nm). All lectins were purchased from Vector Laboratories (BIOKOM, Janki, Poland). WGA is a homodimer of 186 amino acid residues (*M_n_* = 37.51 kDa, PDB entry 1WGT). DBA is a homotetramer of 506 amino acids (*M_n_* = 54.86 kDa, PDB entry 1LU2). WGA and DBA belong to the group of plant legume lectins that specifically recognize carbohydrates containing N-acetylglucosamine (GlcNAc) and N-acetylgalactosamine (GalNAc), respectively. LTL is a homotetramer of 936 amino acids (*M_n_* = 105.18 kDa, PDB entry 2EIG) with binding affinity for L-fucose. Both unlabeled and fluorescently labeled lectins were dissolved in deionized water (dH_2_O) at a concentration of 50 μg/mL (unless otherwise stated).

### 2.3 Surface Silanization

Silanization was conducted in a desiccator from the gas phase. Surfaces were silanized with (3-aminopropyl)­triethoxysilane (APTES, Merck, Poznań, Poland) for 1.5 h.

### 2.4 PDMS Stamps

Two types of PDMS stamps, prepared as in ref 13, were used in the study: flat and patterned. In the latter, it was a linear pattern of 20 μm (elevated region) by 40 μm (grooves) or 20 μm (elevated region) by 20 μm (grooves). The choice of pattern geometries was dictated by two factors: the size of the bladder cancer cells in suspended state and the lack of the passivation of APTES on lectin-patterned substrates. As previously reported,^23^ the diameters of suspended HCV29 and T24 cells are 14.6 ± 2.2 μm and 15.6 ± 3.0 μm, respectively. In addition, the diameter of 5637 cells is 12.0 ± 2.2 μm (n = 100 cells). Considering these cell dimensions, we concluded that lectin stripes narrower than 10 μm would likely be too small to ensure selective cell adhesion, particularly because APTES-passivated regions were not used to suppress nonspecific attachment.

Soft lithography was applied to prepare lectin substrates. Briefly, PDMS stamps were cleaned with a dust-free tissue soaked with 70% ethanol solution (in water) and afterward with dH_2_O. Additionally, the stamps were dried with a stream of N_2_. Next, the stamps were placed in a UV ozone plasma cleaner (L2002A2, Ossila, The Netherlands) working with ambient air for 3 min at RT. Immediately, after the UV ozone plasma treatment, PDMS stamps were covered with 150 μL drop of lectin solution (working concentration of 50 μg/mL) for 15 min. Then, the stamps were rinsed three times with phosphate-buffered saline (PBS, Merck, Poznań, Poland) and gently dried with a stream of N_2_. Then, the stamps were placed on the APTES-modified cover glass slides (Haemacytometer cover glass, Menzel Gläser, Monheim, Germany) and pressed gently for 10–20 s and released. During the contact, lectins were transferred from the surface of a PDMS stamp to the APTES-coated surfaces. Afterward, biopatterned coverslips were placed in a 24-well plate (TPP, Genos, Łódź, Poland), submerged in PBS supplemented with 1% penicillin-streptomycin (1:1 ratio), and stored at 5 °C for further use (for a maximum of 24 h).

### 2.5 Static Adhesion

For static adhesion experiments, the substrates were coated with both unlabeled and fluorescently labeled lectins using the following two protocols. In the former, glass bottoms of the 24-well plates (SensoPlate, Biokom, Janki, Poland) were silanized with APTES, and next, 300 μL of designated fluorescently labeled lectin solution (50 μg/1 mL dH_2_O) was added to each well. After 20 min, lectin solutions were removed, and wells were rinsed 3 times with 300 μL of phosphate-buffered saline. In the latter, lectins were imprinted onto the APTES-functionalized surface of glass coverslips as described above. Regardless of the substrate preparation method, in the next steps, bladder cells were trypsinized and centrifuged for 4 min at 1800 rpm. Afterward, cells were resuspended in the corresponding culture medium (1 mL) and counted. One mL of cell suspension containing 1 × 10^6^ cells/mL was transferred to 15 mL centrifuge tube (TPP, Genos, Łódź, Poland), followed by fluorescently labeling cells with a 10 μM CellTracker Blue CMAC dye (Invitrogen, Thermo Fisher Scientific, Waltham, USA, excitation/emission 353/466 nm). Tubes containing cell suspensions were placed in a CO_2_ incubator for 1 h, then centrifuged, washed in PBS, centrifuged again, and resuspended in culture medium (final cell density 10^4^ cells/mL). Thereafter, 0.5 mL of cell suspension was subsequently deposited on lectin-modified and control surfaces (unmodified glass and APTES-modified surfaces). Next, the samples were incubated in a CO_2_ incubator at 37 °C for 15 min. Next, the substrates were washed in PBS (3×) to remove unbound cells, and the samples were fixed with 3.7% (w/v) paraformaldehyde (PFA, Merck, Poznań, Poland) in PBS for 20 min. At the final step, the samples were visualized using a fluorescence microscope (IX83 Olympus). Images of the cells attached to lectin-coated surfaces were collected in 9 randomly selected locations for each lectin and cell types. The experiment was repeated 3 times.

### 2.6 Cell Patterns

Substrates with lectin patterns were placed in 12-well cell culture plates (TPP, Genos, Łódź, Poland). As references, cells cultured on uncoated and APTES-functionalized substrates were used as controls. 20,000 cells were seeded in each well, and the plates were placed in a CO_2_ incubator for 24 h. Mechanical and morphological characterization was performed only for cells growing on lectin stripes of 20 μm.

### 2.7 Fluorescent Labeling of the Cell Cytoskeleton

For fluorescence, the bladder cancer cells were seeded in wells of 96-well plates (SensoPlate, Biokom, Janki, Poland), whose bottom surfaces were coated with the studied lectins. HCV29, 5637, and T24 cell suspensions were prepared at a density of 15,000 cells per mL of the appropriate culture medium. 100 μL of the cell suspension was added to each well, and the plate was incubated for 24 h. The cells were fixed with a 3.7% PFA solution in phosphate-buffered saline for 20 min. The cell membrane was permeabilized for 5 min using 0.2% Triton X-100 (Sigma-Aldrich, Poznań, Poland) at 4 °C. Then, the samples were blocked with 1% BSA (Sigma-Aldrich, Poznań, Poland) in PBS for 1 h. Microtubules were labeled with antitubulin antibodies 1:500 in PBS (mouse monoclonal anti-α-tubulin antibody; Sigma-Aldrich, Poznań, Poland) overnight at 4 °C and stained with antimouse antibodies conjugated with Alexa Fluor 555 (goat antimouse IgG (H + L) cross-adsorbed secondary antibody, Invitrogen, Thermo Fisher Scientific, Waltham, USA) for 1 h at room temperature. The actin filaments were stained with phalloidin conjugated with Alexa Fluor 488 (1:200 in PBS, Invitrogen, Thermo Fisher Scientific, Waltham, USA) for 45 min. Samples were thoroughly washed with PBS after each step of labeling. In addition to the cytoskeleton, the cell nuclei were stained with Hoechst 34580 (dilution 1:5000 in PBS, Invitrogen, Thermo Fisher Scientific, Waltham, USA).

### 2.8 Fluorescence Microscopy

Fluorescent images were collected by an inverted optical/fluorescence microscope (Olympus IX83) equipped with a set of objectives of various magnifications, a Photometrics Prime BSI Express camera (image resolution 2048 px × 2048 px), a 100 W mercury lamp for fluorescence excitation, and U-FBW and U-FGW filters to record the FITC and TRITC emission. Blue emissions were visualized with a U-FUW filter. Images were recorded at magnifications of 10× and 40×, so the dimensions of the visual field were 1.3 mm × 1.3 mm and 332.8 μm × 332.8 μm, respectively. All images were acquired using CellSens software (Olympus) and processed with ImageJ (version 1.54p, https://imagej.nih.gov/ij/). The experiment was performed in three biological replicates.

### 2.9 AFM Indentation-Relaxation Test

The viscoelastic properties of bladder cells, grown on lectin patterns, were measured by a Nanowizard 4 AFM instrument (Bruker-JPK Instruments, Berlin, Germany) combined with an inverted fluorescence microscope (IX71, Olympus, Japan). The measurements were performed using MLCT BIO–DC cantilevers (Bruker, Berlin, Germany), characterized by a nominal spring constant (*k*_*n*_) of 0.01 N/m and the half-open angle of 35 deg. The cantilevers were calibrated using the thermal tune method.^24^ Measurements were conducted in RPMI-1640 medium supplemented with 1% FBS at RT. AFM measurements were performed in five independent biological replicates.

Force-relaxation maps consisted of individual force curves (i.e., cycles of approach-pause-retract) that were collected over the cell nucleus (2 px × 2 px) from an area of 2 μm × 2 μm. They were acquired at an approach/retract speed of 2 μm/s, with a pause of 6 s. Optical images of cultured cells colocalized with fluorescence images of lectin patterns were used to select cells of interest. For each case, at least 30 cells were probed.

### 2.10 Young’s Modulus Determination

To evaluate the elastic properties of cells, each force curve *F­(z)*, representing a dependence of a load force *F* on the scanner position *z*, was analyzed. In the first step, the approach part of the *F­(z)* curve was converted to a load force *F* and the indentation δ curve^25^ and fitted with the Hertz-Sneddon model, assuming that a four-sided pyramidal tip can be approximated by a cone:

1 $$F\left(\delta \right)=\frac{\tan\alpha }{\sqrt{2}\left(1-\mu^{2}\right)}E\;\delta^{2}$$

where α is the half-open angle of the cone, *E* is the apparent Young’s modulus, and μ is the Poisson’s ratio, set μ to 0.5, as we treat cells as incompressible materials.

### 2.11 Viscoelastic Model

To evaluate the viscoelastic properties of cells, the force curve *F­(z)* was converted to *F­(t)* curve showing a relation between load force and time (*t*). Next, only the part corresponding to the pause was analyzed as it correlates with the force relaxation. The expression, describing this relation, can be obtained from the following equation:

2 $$F\left(t\right)=\frac{\tan\alpha }{\sqrt{2}\left(1-\mu^{2}\right)}E\left(t\right)\;\delta_{0}^{2}$$

In eq 2, *E­(t)* is the elastic relaxation modulus, which describes how viscoelastic materials relax over time when subjected to a constant strain. After taking into account the values of constant parameters (like the Poisson’s ratio and half-open angle of the tip), we obtain the relationship:

3 $$F\left(t\right)=0.495\;E\left(t\right)\;\delta_{0}^{2}$$

where *δ*_0_ is the initial indentation depth obtained from the analysis of the force–distance curves. Regardless of the cell type, the mean initial indentation depth varies from 1.0 to 1.5 μm (see Supporting Information Table S1).

In this study, we utilize the standard linear solid model (SLS) to analyze relaxation curves.^26^ In the SLS model, the viscoelastic properties of the materials are described by three relevant parameters: the relaxation time (*τ*), the equilibrium elastic modulus *E*_inf_ (a parameter quantifying the elastic stiffness of the cell after its viscoelastic relaxation has completed), and the elastic component (*E*_1_) relative to the relaxation process characterized by *τ*. The relation of the parameters is as follows:

4 $$E\left(t\right)=E_{\inf}+E_{1}\;\exp\left(-\frac{t}{\tau }\right)$$

In our study, we fitted to each collected relaxation curve *F­(t)* the following function:

5 $$F\left(t\right)=F_{\inf}+F_{1}\;\exp\left(-\frac{t}{\tau }\right)$$

and sets of *τ, F*_inf_, and *F*_1_ parameters were obtained. *F*_inf_ describes the final state of the deformation process, and *F*_1_ is the relaxation amplitude.^27^ This analysis assumes an instantaneous loading step at the beginning of the hold phase. Since AFM indentation is performed with a finite approach velocity, this approximation may affect the estimation of the short-time viscoelastic response, particularly the *E*_1_ parameter, whereas the long-time modulus (*E*_inf_) is expected to be less sensitive to this effect.^28^ We used eq 2 to recalculate the values of parameters: *F*_inf_ and *F*_1_ to obtain *E*_inf_ and *E*_1_ of eq 4. Examples of fitted curves are shown in Supporting Information (Supporting Information Figure S1).

We also calculated the Deborah numbers (*De*) as

6 $$\mathrm{De}=\frac{\tau }{t_{\mathrm{experiment}}}$$

where *t*_experiment_ = 6 s is the duration of the stress-relaxation experiment and τ is the relaxation time (same in eqs 4 and 5) calculated for each cell. The Deborah number relates the characteristic relaxation time of the material to the observation time and provides a dimensionless measure of whether the viscoelastic response is predominantly solid-like (*De* > 1) or fluid-like (*De* < 1) during the experiment.^15^ Although analysis of the relaxation times alone provides similar information, the Deborah number facilitates interpretation of the viscoelastic response in relation to the experimental time scale. The relaxation period of 6 s was selected to minimize contributions from active cellular remodeling, which has been reported to occur on average after approximately 8 s,^29^ while remaining substantially longer than the characteristic relaxation times measured in the present study (τ = 0.12–4.12 s). Consequently, the observation time exceeded the relaxation time for all cells (*De* < 1), allowing the relaxation process to be captured while reducing the contribution of active cellular responses.

### 2.12 Data Processing, Presentation, and Statistical Analysis

A JPKSPM Data Processing 8.0.166 (Bruker-JPK Instruments), Visual Studio Code (version 1.98), and the Microsoft Python Extension (version 3.10) were used for AFM data processing. The obtained data are presented as median ± median absolute deviation (MAD) unless otherwise stated. For AFM data testing, n refers to the number of cells tested (see Supporting Information Table S2), which is an established approach for this measurement method.^30^ Data sets were tested for outliers using Grubbs’ test (OriginPro 9.1, Gambit). Statistical significance was assessed using the Kruskal–Wallis ANOVA with Dunn’s post hoc test (*p < 0.05; **p < 0.01; ***p < 0.001) at a significance level of 0.05. Statistical analysis was performed with a free trial version of GraphPad Prism 10 software. To quantify the discriminatory power of mechanical and rheological parameters, effect sizes were calculated using Cliff’s delta (Δ), a nonparametric measure of distributional separation (OriginPro 9.1, Gambit). Effect sizes were interpreted as negligible, small, medium, or large according to established thresholds.

## 3 Results

### 3.1 Bladder Cancer Cells Display a Distinct Affinity to DBA, WGA, and LTL Lectins

The affinity of the studied bladder cell lines for selected lectins was investigated using a static adhesion assay. The results, presented in Figure 1, showed no statistically significant differences across the cell lines when treated separately. However, HCV29 cells showed a trend toward lower adhesion on WGA-coated surfaces, whereas both 5637 and T24 cells showed the opposite trend. As both DBA and WGA bind specifically to N-glycans,^6,12^ initially, we have selected DBA and LTL (specific for L-fucose) lectins for further experiments. The static adhesion assay confirmed the affinity of T24 cells for the fucose-binding lectin LTL (Figure 1c), which is consistent with a previous study by Ezeabikwa et al.^6,31^ showing high levels of fucose expression in T24 cells using the *Aleuria aurantia lectin*. LTL pattern-guided adhesion of T24 cells was unsuccessful (Figure S2a). Therefore, WGA patterns were tested instead.

**Figure 1:**
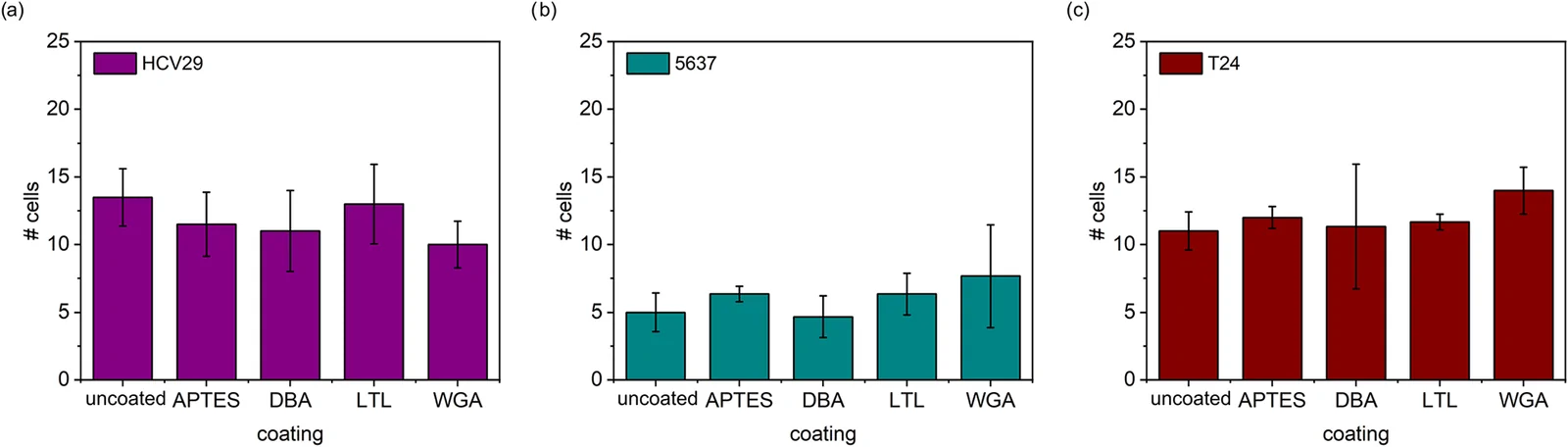
Number of adhered bladder cancer cells HCV29 (a), 5637 (b), and T24 (c) to APTES and lectin-coated glass slides. As a reference, cell adhesion to glass was also investigated. Data are presented as means ± SD, from *n* = 3 biological replicates. Statistical significance was assessed using a Kruskal–Wallis test, and no statistically significant differences in the number of adhered bladder cells were observed on either control or lectin-coated surfaces for each cell line.

### 3.2 Actin Filaments (AF) and Microtubules (MTs) Organization of Bladder Cancer Cells Cultured on a Homogeneously Functionalized Substrates

Figures 2 and S3, and S4 show a distinct organization of actin filaments and MTs in bladder cells cultured on lectin-coated substrates. To quantify these changes, we calculated the cell surface area. The surface area of 5637 cells is smaller than those of HCV29 and T24 cells. The presence of actin bundles is more pronounced in HCV29 and T24 cells than in 5637 cells, as shown in Figure 2a (white arrows). HCV29 and T24 cells have numerous long cell protrusions (filopodia) marked by yellow arrows in Figure 2a. In the case of 5637 cells, the presence of protrusions was not pronounced. The comparison of 5637 cells grown on control substrates and, also, on lectin-coated surfaces reveals that DBA- and LTL-coating induces the formation of longer protrusions in 5637 cells (Figures 2 and S3).

**Figure 2:**
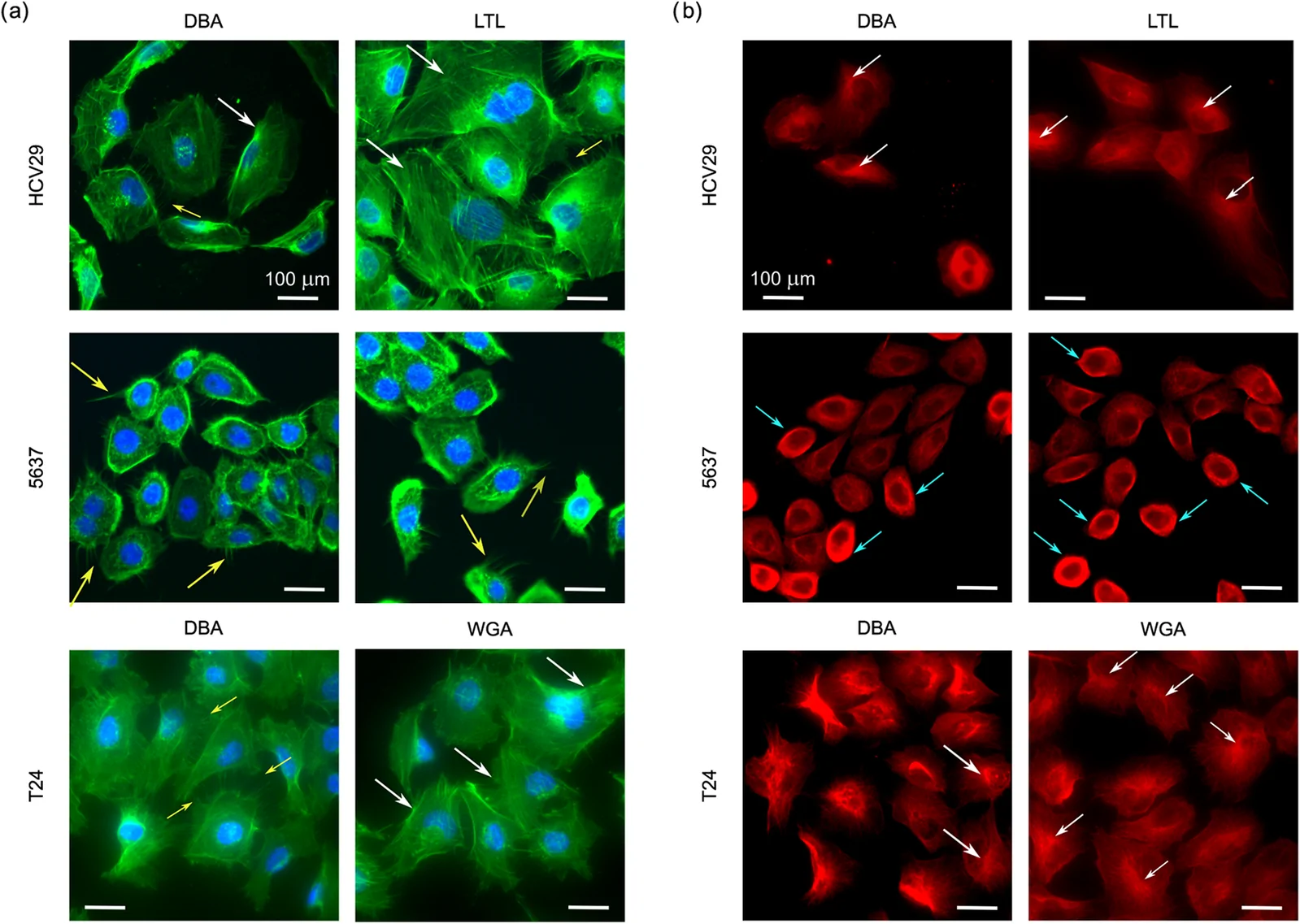
(a) Actin filament organization of HCV29, 5637, and T24 cells cultured on lectin (DBA, LTL, and WGA)-coated substrates. Actin is stained green, and nuclei are stained blue. In panel (a), white arrows indicate cells featuring a large number of actin bundles, while yellow arrows indicate cell protrusions. (b) Corresponding microtubule network. In panel (b), white arrows indicate microtubule-organizing centers (MTOCs), whereas blue arrows indicate circularly organized MTs. Scale bar: 100 μm.

In HCV29 and T24 cells, the MTs spread radially from the nucleus, and the microtubule-organizing centers (MTOCs) are usually clearly visible (Figure 2b, white arrows). In 5637 cells, MTs are mostly densely packed and tightly encircle the nucleus, but sometimes form a network of elongated parallel fibers across the cell body (higher fluorescence intensity, Figure 2b).

Fluorescence staining of 5637 cells cultured on lectin-coated surfaces revealed long cell protrusions with no change in cell morphology. This contrasts with 5637 cells cultured on glass, where only a few short protrusions were observed (see Supporting Information Figure S3). In HCV29 and T24 cells cultured on lectin-modified substrates, numerous actin bundles formed, with microtubules (MTs) organizing radially from the cell nucleus.

### 3.3 Mechanical and Rheological Properties of Bladder Cancer Cells Cultured on Lectin-Patterned Substrates

Our goal was to verify whether wide lectin-patterned substrates, when used as platforms for culturing bladder cells, would influence the cells’ mechanical and rheological properties. Figure 3a–c shows a schematic overview of the fabrication process for lectin-patterned surfaces, which were subsequently used for culturing bladder cells (Figure 3d,e) and characterizing their mechanical properties (Figure 3e,f).

**Figure 3:**
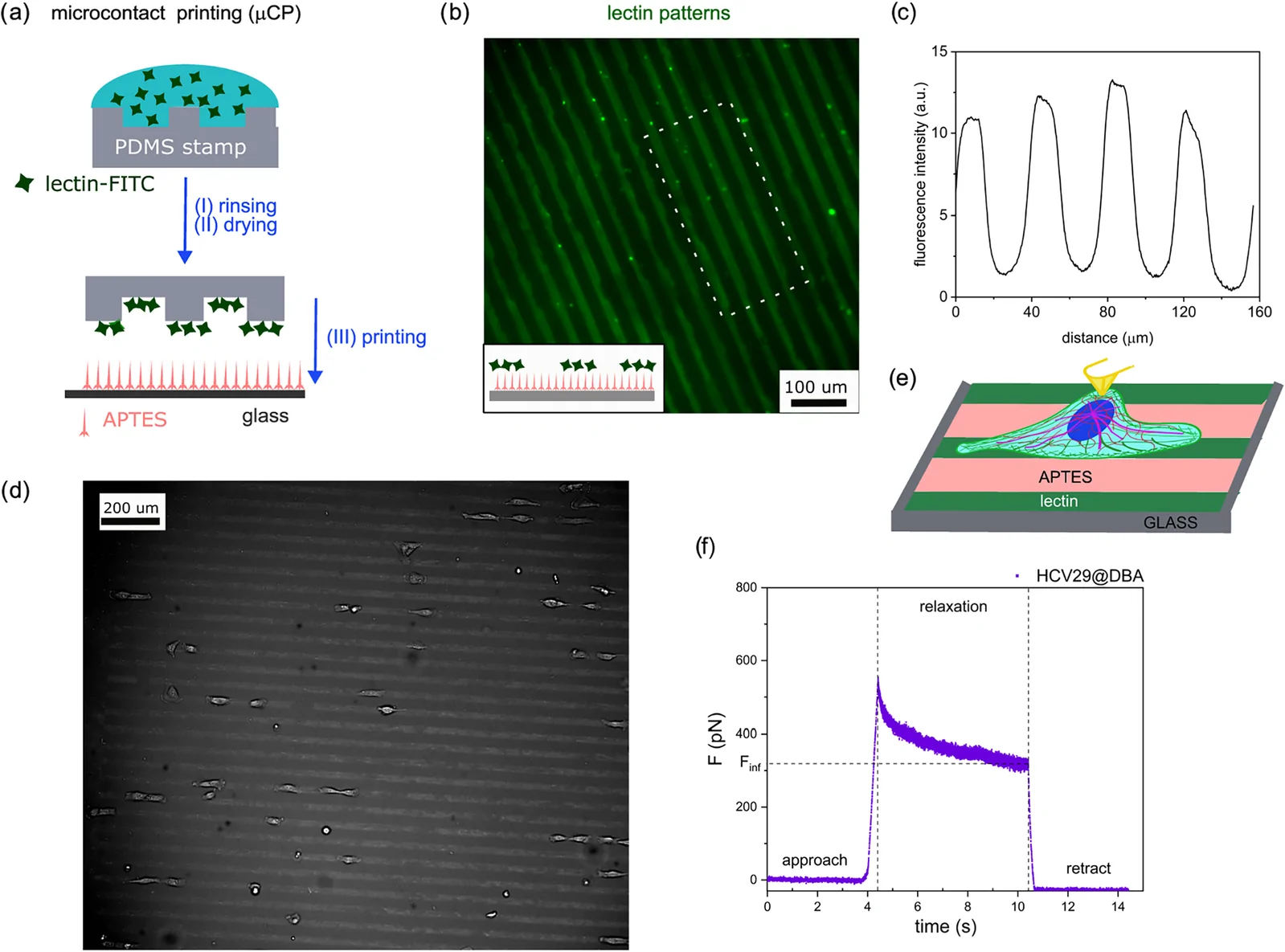
(a) Scheme showing the fabrication of lectin patterns by the microcontact printing method (μCP). (b) Example of a fluorescence microscopy image showing periodic lectin patterns of 20 × 20 μm. (c) Fluorescence intensity profile obtained for the selected image area in panel (b); white dashed lines indicate the area. (d) Greyscale fluorescence microscopy image of DBA-FITC lectin patterns with HCV29 cells. (e) Graph showing the AFM probe indenting a cell with an elongated shape imposed by the chemical substrate pattern. (f) Example of a single cycle of the AFM indentation-relaxation test performed on an HCV29 cell grown on the striped DBA pattern.

We have calculated the apparent Young’s modulus values for cells growing on linear lectin patterns (stripes) from the approach part of the force–distance curves. From the relaxation part of the force-time curves (see Figure 3f for an example), we obtained parameters describing the viscoelastic properties of cells, *E*_inf_ and *De*. The results are discussed in the following subsections.

#### 3.3.1 Determination of the Elastic Properties of Patterned Bladder Cancer Cells from Classical Force-Spectroscopy Experiments

The striped biopattern of the substrate induces changes in the shape of the bladder cancer cells (Figures 4d–f and 7). The cells fit the biopattern and become more elongated (Figures 4d–f and 7). This morphological modification is accompanied by changes in the mechanical properties of the studied cells (Figure 4a–c and Table S3). In Figure 4a–c, we compare the apparent Young’s moduli obtained for HCV29, 5637, and T24 cells cultured on reference (uncoated glass and amino-functionalized glass substrates) and lectin-patterned substrates.

**Figure 4:**
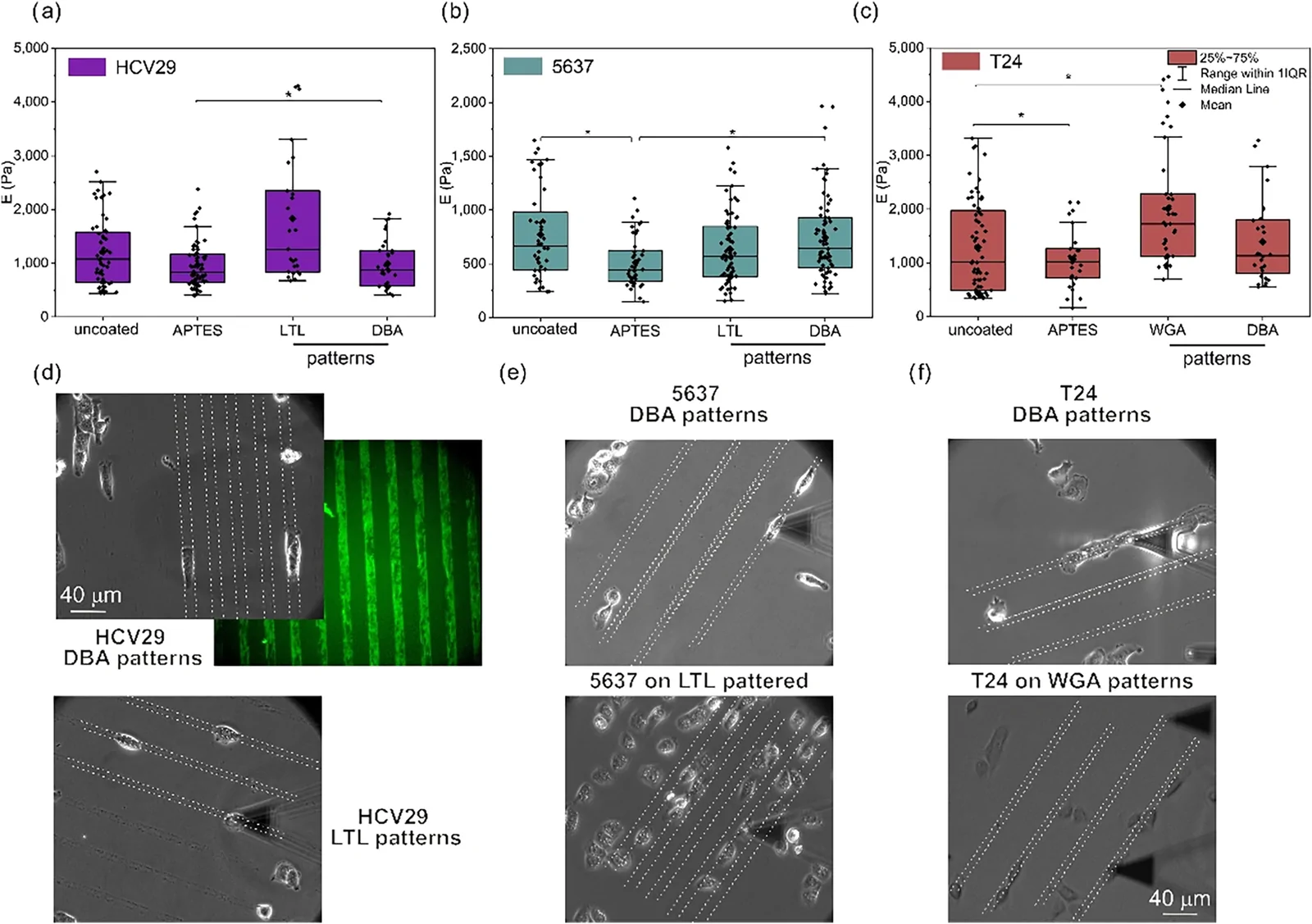
Apparent Young’s modulus (*E*) obtained for HCV29 (a), 5637 (b), and T24 (c) cells cultured on homogeneous glass and APTES-functionalized substrates and lectin patterns (LTL, DBA, and WGA stripes). Data are shown as box plots (median, interquartile range, and whiskers) with overlaid individual data points. Statistical significance was assessed using the Kruskal–Wallis ANOVA with Dunn’s post hoc test (*p < 0.05; **p < 0.01; ***p < 0.001). (d–f) Optical images (saved during the AFM measurement) of bladder cancer cells on DBA, LTL, and WGA patterned substrates. In panel (d), a fluorescence image of the lectin pattern (labeled in green) is shown. Dotted white lines mark the borders of lectin stripes in the optical images of HCV29, 5637, and T24 cells on lectin patterns.

For homogeneous surfaces, we observed softening of 5637 cells when cultured on the APTES-functionalized glass surface. The elastic modulus determined for 5637 cells drops from 664 ± 234 Pa (*n* = 54 cells, on glass) to 440 ± 132 Pa (*n* = 66 cells, on APTES). For T24 cells, statistical analysis revealed differences in the distributions of *E* for T24 cells grown on glass and APTES surfaces.

In the case of biopatterned substrates, data presented in Figure 4a show that the elastic modulus of HCV29 cells grown on DBA patterns is 879 ± 315 Pa (*n* = 36 cells), which is higher than that of cells grown on the APTES-modified substrates but not different from the modulus for HCV29 cells grown on the glass surface. In the case of 5637 cells, there is no statistically significant difference in the *E* of these cells cultured on the glass or lectin-patterned substrates (664 ± 234 Pa (*n* = 44 cells), 642 ± 215 Pa (*n* = 71 cells), and 566 ± 220 Pa (*n* = 72 cells) cultured for glass surface, DBA and LTL patterns, respectively). For T24 cells, the highest value of the apparent Young’s modulus (1725 ± 598 Pa, *n* = 39 cells) was observed for cells grown on the striped WGA pattern. T24 cells cultured on the striped DBA pattern are characterized by an *E* = 1133 ± 480 Pa (*n* = 28 cells). It should be noted that while HCV29 cells grow on striped lectin patterns, 5637 cells grow on APTES between the lectin stripes (Figures 3d, 4d, 7, and S2).

T24 cells do not show such selectivity and grow randomly on the patterned substrate (see Supporting Information Figure S2 for an example). However, there are still areas of lower cell density where some T24 cells bind, adhere, and proliferate on the lectin stripes (as probed by AFM), and as a result, their morphology is altered (Figures 5f, 7, and S2).

**Figure 5:**
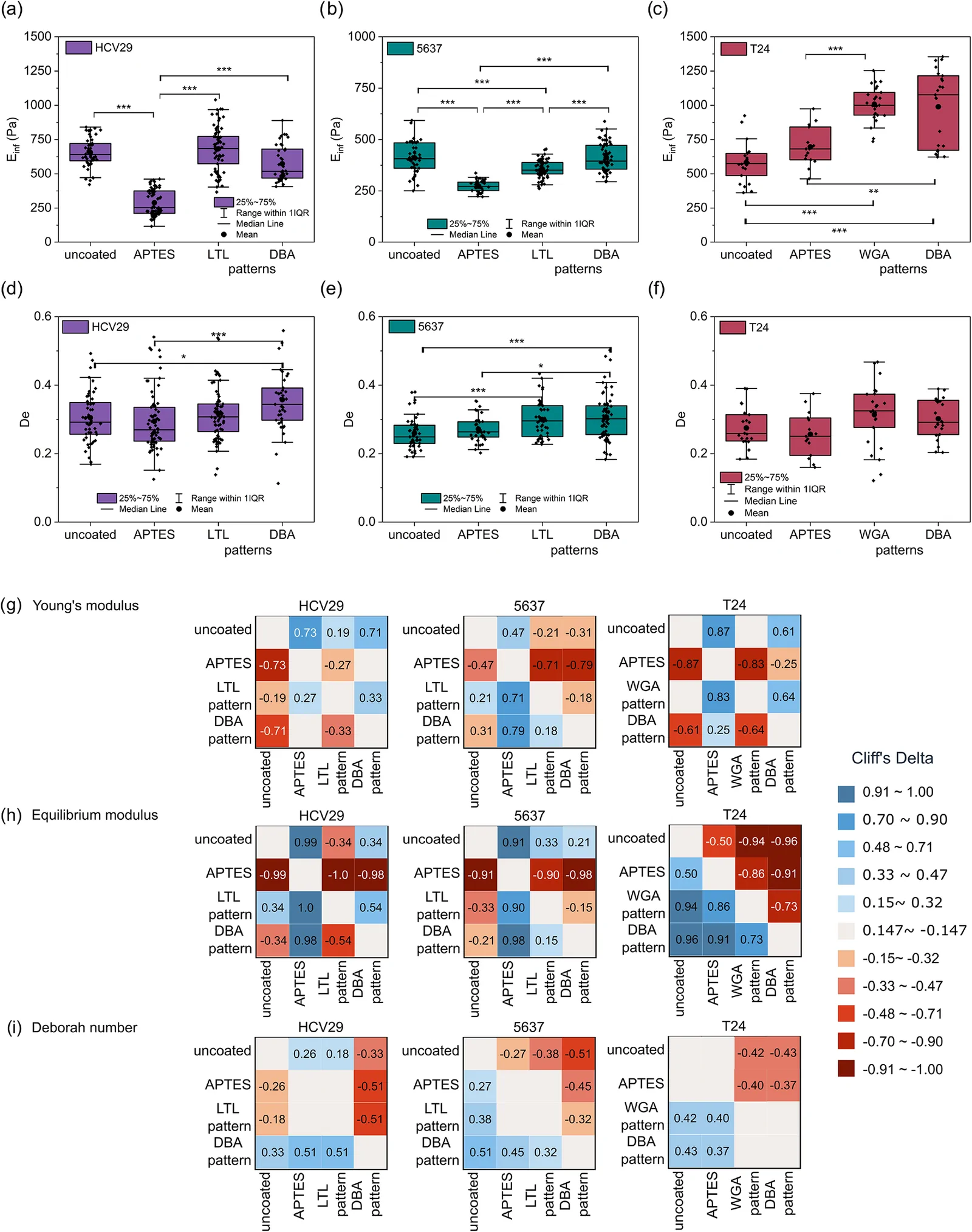
(a–c) Equilibrium elastic modulus (*E*_inf_), and (d–f) Deborah numbers (*De*) obtained for HCV29, 5637, and T24 cells cultured on different substrate conditions (uncoated, APTES, LTL/WGA, and DBA-patterned surfaces). Data are shown as box plots (median, interquartile range, and whiskers) with overlaid individual data points. Statistical significance (Kruskal–Wallis ANOVA with Dunn’s post hoc test) between groups is indicated (*p* < 0.05, *p < 0.01, **p < 0.001). (g–i) Heatmaps of Cliff’s delta (Δ) representing pairwise nonparametric effect sizes between substrate conditions for each cell line and mechanical parameter (Young’s modulus, equilibrium modulus, and Deborah number). Positive Δ values indicate higher measurements in the row condition, whereas negative values indicate higher measurements in the column condition. The magnitude of Δ reflects the degree of distributional separation (|Δ| → 0, overlap; |Δ| → 1, complete dominance), and numerical values denote exact coefficients.

The obtained data show that chemical and geometrical modifications of the substrate can induce stiffening of bladder cancer cells.

#### 3.3.2 Determination of the Elastic Properties of Patterned Bladder Cancer Cells from Stress-Relaxation Experiments

We also compare changes in the equilibrium elastic modulus (*E*_inf_), which accounts for the solid-like response of viscoelastic materials, which approaches 0 for fluids. The obtained *E*_inf_ are presented in Figure 5a–c (see also Sup. Tab. S4). The pronounced changes in the equilibrium elastic modulus were obtained for 5637 and T24 cells. A statistically significant difference (*p* < 0.001) was found for almost all sample type combinations. The lowest modulus, 272 ± 22 Pa (*n* = 41), was found for 5637 cells growing on a homogeneous APTES-modified substrate. The highest *E*_inf_ was observed for DBA-based patterns, showing that the elastic properties of HCV29 cells grown on DBA-patterned and glass surfaces are similar at the relaxation equilibrium. For 5637 cells grown on LTL patterns, a drop in *E*_inf_ is observed compared to the elastic properties of these cells grown on a glass surface (352 ± 30 Pa vs 407 ± 53 Pa, respectively). In the case of HCV29 cells, the lowest elastic equilibrium moduli were observed for cells grown on DBA patterns and a homogeneous APTES-functionalized glass surface. HCV29 cells became stiffer when grown on glass or striped LTL patterns.

The highest *E*_inf_ values were found for T24 cells grown on striped lectin patterns. For T24 cells cultured on reference substrates (glass and APTES-functionalized surfaces), *E*_inf_ dropped to approximately 630 −700 Pa. The rheological data revealed that HCV29 and 5637 cells grown on the APTES substrate exhibit a more fluid-like behavior (the lowest values of *E*_inf_).

#### 3.3.3 Lectin Patterns Induced Changes in the Viscosity of Bladder Cancer Cells

Substrate biopatterns induce changes not only in the mechanical properties of the studied cells but also in their fluidity, quantified by the Deborah number (Figure 5d–f). The lower the *De*, the more fluid-like the behavior of the cell. HCV29 cells cultured on APTES-functionalized glass substrates are characterized by the lowest *De*, i.e., 0.270 ± 0.038 (*n* = 54). The *De* obtained for HCV29 cells cultured on striped DBA and LTL patterns indicates more solid-like cell characteristics when compared to the reference samples. The highest *De* (0.344 ± 0.046, n = 36) was found for nontumorigenic immortalized urothelial cells grown on a striped DBA pattern (Figure 5d, Supporting Information Table S5). 5637 cells behaved similarly to HCV29 cells (Figure 5e, Supporting Information Table S5). *De* of 0.302 ± 0.043 (*n* = 71) was found for 5637 cells growing on striped DBA patterns. For the control substrates (glass vs APTES-functionalized glass), there is no statistically significant difference in *De*, i.e., 0.249 ± 0.026 (*n* = 44) vs 0.264 ± 0.022 (*n* = 41). 5637 cells growing on LTL: APTES and DBA: APTES patterns revealed more solid-like behavior than those cultured on the glass.

Figure 5f shows the Deborah numbers obtained for T24 cells. No statistical significance was observed in changes to the fluidity parameter (*De*). Although T24 cells cultured on lectin patterns showed a slight tendency toward higher *De* values, this trend should be interpreted with caution due to the lack of statistical significance (see Table S5).

A comparison of the fluidity parameters indicates that HCV29 and 5637 cells grown on DBA biopatterns exhibit more solid-like behavior than cells grown on reference surfaces. In contrast, the fluidity of T24 cells appears largely unaffected by culture conditions (i.e., substrate pattern).

### 3.4 Substrate-Dependent Modulation of Viscoelastic Phenotype in Bladder Cancer Cells

Employing two approaches to study the mechanical properties of bladder cells resulted in quantifying their viscoelastic properties by parameters: the apparent Young’s modulus, equilibrium modulus, and Deborah number. The *E* indicates how easily the cells can be deformed, whereas *E*_inf_ characterizes the long-term elastic behavior of cells,^29^ and *De* indicates fluidity of cells. In Figure 5g–i, we present heatmaps of Cliff’s delta (Δ) showing the separation of data distribution as a function of substrate modification for HCV29, 5637, and T24 cells.

Substrate modification induced pronounced, cell-line-dependent changes in the viscoelastic properties of bladder cells. While Young’s modulus exhibited moderate variability across conditions, *E*_inf_ and *De* revealed substantially stronger and more consistent differences. In particular, *E*_inf_ showed large effect sizes across multiple substrate comparisons, with several pairwise Cliff’s Δ values approaching unity, indicating near-complete separation of distributions. These effects were most prominent for the T24 and 5637 cell lines, whereas HCV29 cells exhibited comparatively smaller changes.

Across substrates, distinct mechanical profiles emerged, with patterned surfaces (LTL/WGA and DBA) generally associated with increased equilibrium stiffness relative to that of uncoated and APTES-modified surfaces. These shifts were accompanied by corresponding changes in the Deborah number, suggesting that substrate chemistry and pattern influence not only stiffness but also the balance between elastic and viscous behavior. Notably, Deborah number differences were more subtle than those observed for *E*_inf_, yet they still indicated substrate-dependent modulation of time-dependent mechanical response for HCV29 and 5637 cells.

Collectively, these results demonstrate that cell–substrate interactions regulate viscoelastic phenotype, with equilibrium modulus providing the most sensitive descriptor of substrate-induced effects.

Figure 6 compares viscoelastic properties between bladder cell lines (HCV29, 5637, and T24) cultured on identical substrates, revealing substrate-dependent differences in intercell-line relationships. For Young’s modulus (Figure 6a–c), all substrates show a consistent trend with T24 exhibiting higher stiffness compared to HCV29 and 5637, although the magnitude of these differences varies across conditions. Effect size analysis (Cliff’s Δ) indicates moderate to strong separation, particularly between T24 and 5637. More pronounced differences are observed for *E*_inf_, where substrate modification significantly amplifies separation between cell lines (Figure 6d–f). On APTES and DBA-patterned substrates, effect sizes approach complete dominance (|Δ| ≈ 1) for several comparisons, indicating minimal overlap between distributions. In contrast, uncoated substrates exhibit weaker, though still significant, differences. Simultaneously, *De* (Figure 6g–i) shows less consistent differences between cell lines. While some statistically significant changes are observed, particularly on uncoated and DBA-patterned substrates, effect sizes are generally low to moderate, indicating substantial overlap between distributions.

**Figure 6:**
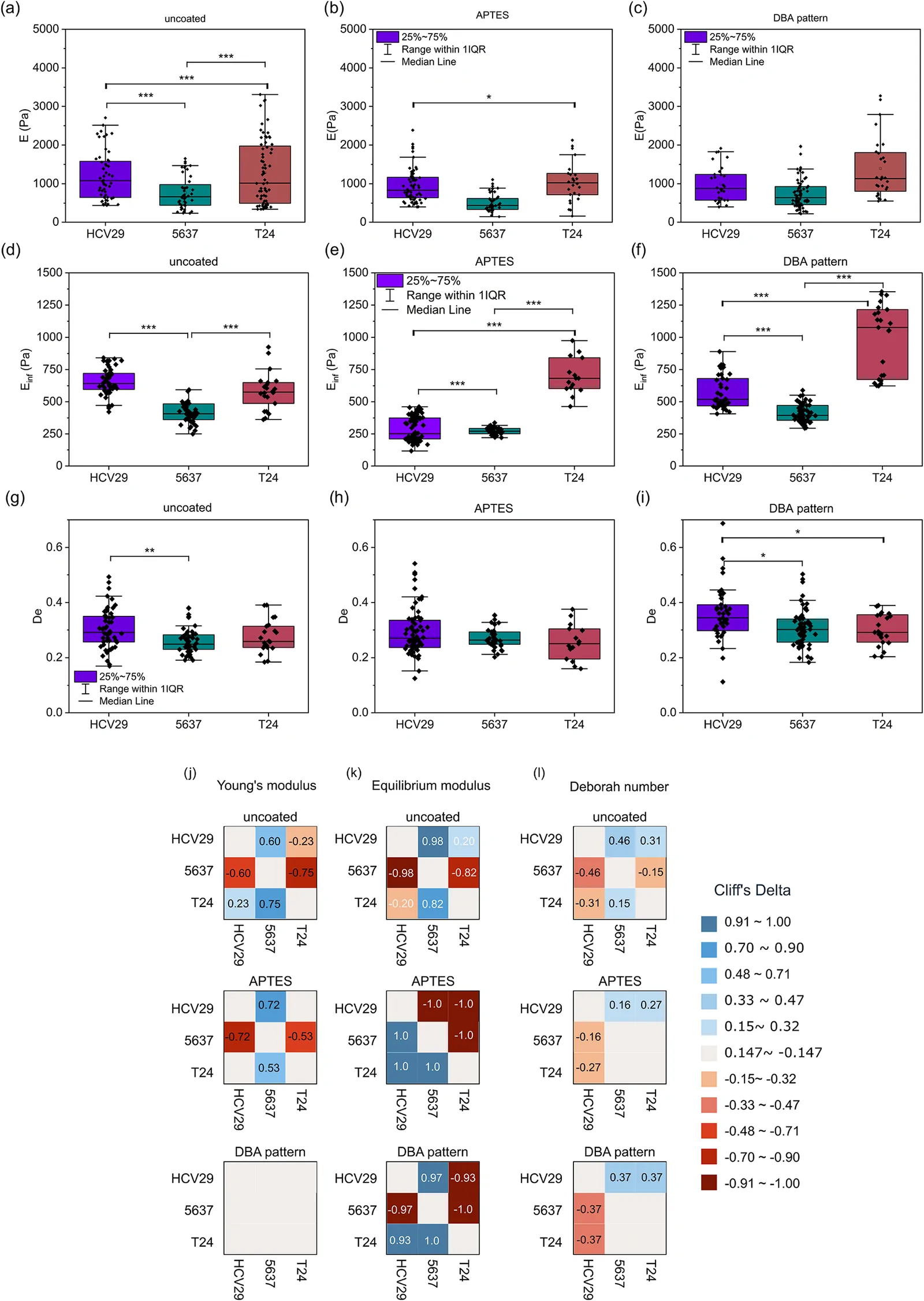
Mechanical and rheological characterization of three cell lines (HCV29, 5637, and T24) cultured on different substrate conditions (uncoated, APTES, and DBA-patterned surfaces). (a–c) Young’s modulus (*E*), (d–f) equilibrium modulus (*E*_inf_), and (g–i) Deborah number (*De*) presented as box plots (median, interquartile range, and whiskers) with overlaid individual data points. Statistical significance between groups is indicated (*p* < 0.05, *p < 0.01, **p < 0.001). (j–l) Corresponding heatmaps of Cliff’s delta (Δ) showing pairwise nonparametric effect sizes between cell lines for each substrate and mechanical parameter. Positive Δ values indicate higher measurements in the row group, whereas negative values indicate higher measurements in the column group. The magnitude of Δreflects the degree of distributional separation (|Δ| → 0, overlap; |Δ| → 1, complete dominance). Numerical values denote exact Δ coefficients.

Importantly, the relative differences between cell lines are not invariant across the substrates. While general trends (e.g., higher stiffness of T24 cells) are partially preserved, the magnitude and strength of separation vary depending on the substrate chemistry and patterning. This is particularly evident for *E*_inf_, where substrate modification reshapes intercell-line relationships, leading to either moderate separation or near-complete dominance depending on the condition.

Overall, these results demonstrate that substrate modification not only influences absolute mechanical properties but also modulates the relative viscoelastic phenotype between cell lines, highlighting a strong cell line–substrate interaction.

### 3.5 Reorganization of the Cell Cytoskeleton Driven by the Geometry and Physicochemical Properties of the Lectin Patterns

Figure 7 presents examples of fluorescence images showing the organization of actin filaments and MTs in bladder cells growing on striped lectin patterns. The organization of the cytoskeleton in HCV29 cells growing on LTL and DBA patterns is shown in Figure 7a,b. In the case of the DBA pattern, the change in HCV29 cell shape is more pronounced. The cell body is elongated (white arrow in Figure 7b), and its width corresponds to the lectin stripe, so the organization of the MTs is no longer radial, but they are arranged along the cell body parallel to the DBA stripes.

**Figure 7:**
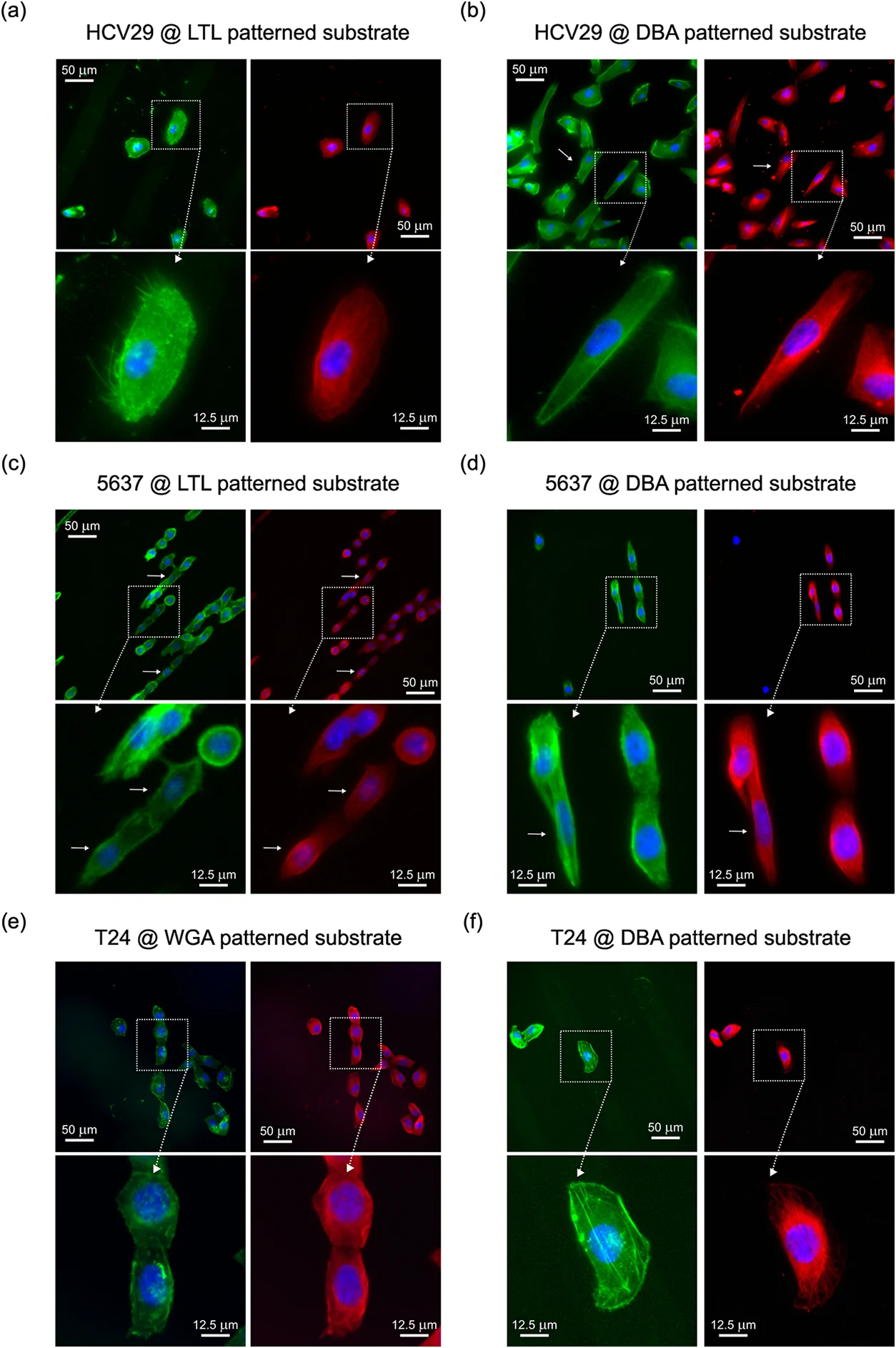
Fluorescence images of actin fibers and microtubule organization of HCV29 (a, b), 5637 (c, d), and T24 (e, f) cells cultured on lectin patterns. Actin is stained in green, MTs in red, and nuclei in blue. White arrows point to cells, showing the elongated cell body shape imposed by the substrate patterns. The cells edged by white squares are zoomed and presented in the lower rows of the panels. HCV29 (a, b) and T24 (e, f) grow on lectin-coated stripes, while 5637 cells (c, d) grow on APTES stripes (between lectin-covered stripes). During the imaging of actin filaments, FITC-stained lectin patterns were bleached. Weak fluorescence of the lectin patterns is visible in panels (a) and (f).

In the case of LTL stripes, HCV29 cells also adapt to the width of the pattern. T24 cells behave quite similarly to HCV29 cells (compare Figure 7f,a). The most intriguing result is shown in Figure 7c,d, which shows aligned 5637 cells. Regardless of the lectin type, they adhere and proliferate on APTES stripes between the striped lectin patterns. White arrows in Figure 7c point to elongated 5637 cells in which MTs are organized longitudinally. Fluorescence staining of bladder cells grown on striped patterns revealed pronounced changes in the cytoskeleton organization and cell morphology. The pattern imposes an elongated morphology on the cells studied.

## 4 Discussion

The concept of applying lectin microarrays for cell capture and positioning in cell screening and biosensor design was proposed years ago.^10^ However, the utility of such biopatterns remains limited due to a lack of understanding of how lectin-functionalized surfaces modulate the functional properties of cancer cells, such as their proliferation, motility, cytoskeletal architecture, and viscoelastic properties. In a recent paper, Luty et al.^21^ demonstrated that WGA exerted the strongest effects among the lectins tested (DBA, WGA, *Lens culinaris agglutinin* (LCA), and *Phaseolus vulgaris leucoagglutinin* (PHA-L)), particularly in invasive T24 cells, increasing proliferation while reducing migration and causing ∼ 60% cell stiffening. These findings indicate that, although lectins are valuable tools for cancer detection due to their ability to recognize aberrant glycosylation patterns, they actively modulate cancer cell behavior rather than acting solely as passive diagnostic tools. This dual role suggests that lectin-induced cellular changes may affect diagnostic reliability and should be therefore carefully considered when developing lectin-based biosensors or biopatterns for high-throughput AFM-based mechanocharacterization.

Although static adhesion assays showed comparable initial adhesion of HCV29, 5637, and T24 cells to control (glass, APTES) and lectin-coated substrates (LTL/WGA, DBA), cell behavior on patterned substrates may still lead to distinct positioning outcomes, as demonstrated in this study. This possibility is further supported by changes in cytoskeletal organization observed in cells cultured on uniformly lectin-coated substrates. Qualitative analysis of fluorescence images revealed lectin-dependent alterations in actin organization, cell protrusions, and microtubule arrangement. This result agrees with a recent report by Luty et al.^21^ In parallel, the work by Rigato et al.^14^ showed that changes in fibronectin (an ECM protein) pattern geometry alone lead to alterations in focal adhesion distribution and local variations in Young’s modulus. Considering these findings, in this work, we address the gap in understanding whether substrate confinement and lectin chemistry jointly modulate the viscoelastic properties of cells.

Classical AFM analysis based on the apparent Young’s modulus revealed only minor changes in the mechanical properties of cells cultured on lectin biopatterns compared with control cells grown on APTES. In contrast, viscoelastic analysis provided substantially greater discrimination between experimental conditions, despite the limitation that the present viscoelastic analysis assumes an instantaneous loading step. Because AFM indentation is performed with a finite loading rate, the short-time relaxation response may be partially completed during the loading phase, potentially affecting estimation of the *E*_1_ parameter. Previous work has shown that finite loading times primarily influence short-time viscoelastic parameters, whereas long-time parameters are less affected.^28^ Therefore, the biological interpretation in this study was based primarily on the equilibrium elastic modulus, which reflects the long-term elastic response and emerged as the most sensitive mechanical descriptor.

The 6 s relaxation period was selected as a compromise between providing sufficient time to capture the passive viscoelastic relaxation of the cells and minimizing the contribution of active cytoskeletal remodeling, which has been reported to occur, on average, after approximately 8 s. Consistent with this rationale, the calculated Deborah numbers were below unity, indicating that the experimental observation time exceeded the characteristic relaxation times of the cells. Nevertheless, occasional force-relaxation curves exhibited signatures of active mechanical responses within the 6 s observation window, suggesting that the transition between passive viscoelastic relaxation and active cellular adaptation is gradual rather than abrupt.

For all studied cell lines, cells cultured on lectin stripes exhibited increased *E*_inf_ values relative to homogeneous APTES coatings. This observation was further supported by effect size analysis using Cliff’s delta (Δ), which showed consistently larger and more robust differences for *E*_inf_ than for *E*. In several cases, Δ values for *E*_inf_ approached unity, indicating near-complete separation of distributions and minimal overlap between conditions, whereas effect sizes for Young’s modulus were smaller and more variable. These findings highlight the equilibrium modulus as the most discriminative parameter for distinguishing cell responses to lectin-mediated confinement and underscore the importance of time-dependent mechanical descriptors in cell mechanophenotyping.

Changes in cell fluidity were also observed on lectin biopatterns, particularly for HCV29 and 5637 cells grown on DBA-striped patterns (Figure 5 and Table S7). This finding is interesting in light of the static adhesion results and literature data indicating lower affinity of these cell lines for N-glycans.^6^ Furthermore, 5637 cells preferentially adhered to APTES-functionalized regions between lectin stripes, whereas HCV29 cells predominantly adhered to the lectin-coated stripes. These observations suggest that, particularly for 5637 cells, geometric confinement may play a more dominant role than lectin affinity in guiding cell positioning and subsequent viscoelastic adaptation, further highlighting the cell line-specific nature of mechanosensitive responses.

The greater sensitivity of the equilibrium modulus suggests that the observed changes are associated primarily with long-term adaptive cellular processes rather than with immediate elastic responses. Therefore, the altered viscoelastic behavior is unlikely to result from solely lectin-mediated glycan interactions. Instead, lectins likely serve as initial adhesion and positioning cues, while the final mechanical phenotype reflects the combined effects of geometric confinement, cell mechanosensing, cytoskeletal reorganization, and cell-driven remodeling processes, including ECM deposition.^32^ This interpretation is further supported by fibronectin staining (Supporting Information Figure S5), which revealed pronounced cell-line-dependent differences in ECM production, with low fibronectin deposition in 5637 cells, moderate deposition in T24 cells, and high deposition in HCV29 cells. The spatial confinement imposed by micropatterns likely promotes cell alignment and anisotropic spreading, which may enhance actin cytoskeleton tension and focal adhesion maturation.

The sensitivity of the invasive T24 cells to geometric lectin patterns was low. The majority of cells adhered randomly and did not adapt to the lectin patterns (Supporting Information Figure S2). The low sensitivity of T24 cells to lectin biopatterns, which we observed here, can be related to their invasive phenotype, which is characterized by high migratory potential and short doubling time compared to HCV29 and grade III (HT1376) cells.^5,21,31^ In addition, in our previous work, we showed by single-cell force spectroscopy that detachment of T24 cells from WGA layer requires much lower energy than for HCV29 cells,^12^ which also can be a fingerprint of the invasiveness potential. Mehanna et al. showed that high metastatic potential cells (data for various cancer types) are more aggressive through detachment rather than migration.^33^ In addition, Luty et al.^21^ compared transmigration and migration of T24 cells on lectin-coated surfaces (including WGA and DBA). Their data show that WGA coating diminishes migration of T24 cells, this can relate to the fact that WGA is a homodimer with eight binding sites,^34^ while DBA has only four binding sites,^35^ so the difference in lectin valency can relate to difference in migration of T24 cells on homogeneous DBA- and WGA-coatings.

Fluorescence imaging revealed elongation of cells grown on lectin patterns. Analysis of the aspect ratio and roundness of HCV29 and 5637 cells grown on LTL-coated and LTL-patterned surfaces, shown in Supporting Information Figure S6, confirmed quantitatively this observation. Actin stress fibers in HCV29 and T24 cells appeared to follow the direction of the DBA stripes (Figure 7). A similar qualitative trend was observed for the microtubule network in cells grown on striped lectin patterns. These observations support a direct link between geometry-induced cytoskeletal organization and the observed shift toward a more solid-like viscoelastic behavior, what is consistent with previous studies showing that cytoskeletal rearrangement and cell polarization—induced by elasticity gradients, micropatterns, or cytoskeleton-targeting drugs—can lead to either stiffening or softening of cells.^18,29,36–42^

Importantly, our results demonstrate that high lectin affinity alone does not dictate lectin-guided cell positioning or mechanical adaptation. Instead, cell-specific responses emerge from the interplay between glycan-mediated adhesion and confinement-induced mechanoadaptation. While the present study focused on selected lectins and pattern dimensions, future investigations should explore a broader range of geometries, lectin specificities, other cancer cell lines, glycan profiling of cells, and rheological testing conditions—including varying relaxation times and applied loads—to further refine mechanophenotypic discrimination. Also, a limitation of the present study is that the individual contributions of lectin chemistry and geometric confinement could not be fully disentangled.

## 5 Conclusions

In this work, using lectin-functionalized homogeneous and micropatterned substrates, we demonstrate that lectin-mediated adhesion cues and geometric confinement jointly modulate cell morphology, cytoskeletal organization, and nanoscale mechanical behavior of bladder cells. Striped lectin patterns promoted cell elongation and cytoskeletal polarization, accompanied by a shift toward a more solid-like viscoelastic phenotype. Importantly, viscoelastic parameters, particularly the equilibrium elastic modulus, provided greater sensitivity in distinguishing mechanical phenotypes of bladder cancer cells than the apparent Young’s modulus alone, highlighting the importance of time-dependent mechanical descriptors in cancer cell mechanophenotyping. Our findings further suggest that the observed mechanical changes are not driven solely by lectin–glycan interactions but arise from the combined effects of initial adhesion cues, geometric confinement, mechanosensing, and subsequent cell-driven remodeling processes, including ECM deposition. Finally, invasive T24 cells exhibited limited responsiveness to the patterned substrates, indicating that highly metastatic cells may be less susceptible to lectin-guided positioning strategies.

## Supplementary Material



## Notes

Mean indentation depths (Table S1), representative AFM stress-relaxation curves with model fits (Figure S1), number of analyzed cells (Table S2), fluorescence images of cell positioning on lectin micropatterns (Figure S2), actin and microtubule organization on homogeneous substrates (Figures S3–S4), fibronectin staining protocol and representative fluorescence images (Figure S5), quantitative cell morphology analysis (Figure S6), Young’s modulus (Table S3), equilibrium elastic modulus (Table S4), Deborah numbers (Table S5), and fluorescence intensity profiles confirming the lectin micropattern quality (Figure S7) (PDF)

## Abbreviations

AF: actin filaments
AFM: atomic force microscopy
APTES: (3-aminopropyl)­triethoxysilane
DBA: *Dolichos biflorus agglutinin*
FBS: fetal bovine serum
HEPES: 4-(2-hydroxyethyl)­piperazine-1-ethanesulfonic acid
LTL: *Lotus tetragonolobus lectin*
MTOC: microtubule-organizing center
MTs: microtubules
PBS: phosphate-buffered saline
PDMS: polydimethylsiloxane
PFA: paraformaldehyde
RT: room temperature
SLS: standard linear solid model
μCP: microcontact printing
WGA: *Wheat germ agglutinin*
