# Supplementary material for: Lectin-Micropatterned Biointerfaces Regulate Cytoskeletal Organization and Viscoelastic Phenotypes of Bladder Cancer Cells
Source: ACS Omega. 2026 Aug 6;11(32):48215–29. doi: 10.1021/acsomega.6c04675 (PMC13491469; doi:10.1021/acsomega.6c04675)
Supplement: Supplementary file 1 [file ao6c04675_si_001.pdf]

Supplementary Materials for

Lectin-micropatterned biointerfaces regulate cytoskeletal organization and viscoelastic phenotypes of bladder cancer cells

Joanna Zemła [1,], Justyna Śmiałek-Bartyzel [1], Bartosz Brzuchacz [2] and Małgorzata Lekka [1]*

[1] Institute of Nuclear Physics, Polish Academy of Sciences, PL-31342 Krakow, Poland

[2] Faculty of Chemical Engineering and Technology, Cracow University of Technology, Warszawska 24, PL-30155 Krakow, Poland

* Correspondence: e-mail: joanna.zemla@ifj.edu.pl; Tel.: 0048 12 662 8062

Table S1. Mean maximum indentation depth (δ_{max}) during relaxation measurements of bladder cells cultured on reference substrates (homogenous glass and APTES) and striped lectin patterns. Data presented as means ± SD.

δ_{max} (*µm*)	*HCV 29*	*T24*	*5637*
glass	1.24 ± 0.42	1.08 ± 0.37	1.16 ± 0.43
APTES	1.17 ± 0.35	1.02 ± 0.37	1.45 ± 0.33
striped DBA patterns	1.21 ± 0.51	0.95 ± 0.33	1.17 ± 0.36
striped LTL patterns	1.17 ± 0.43	-	1.19 ± 0.35
striped WGA patterns	-	1.01 ± 0.31	-

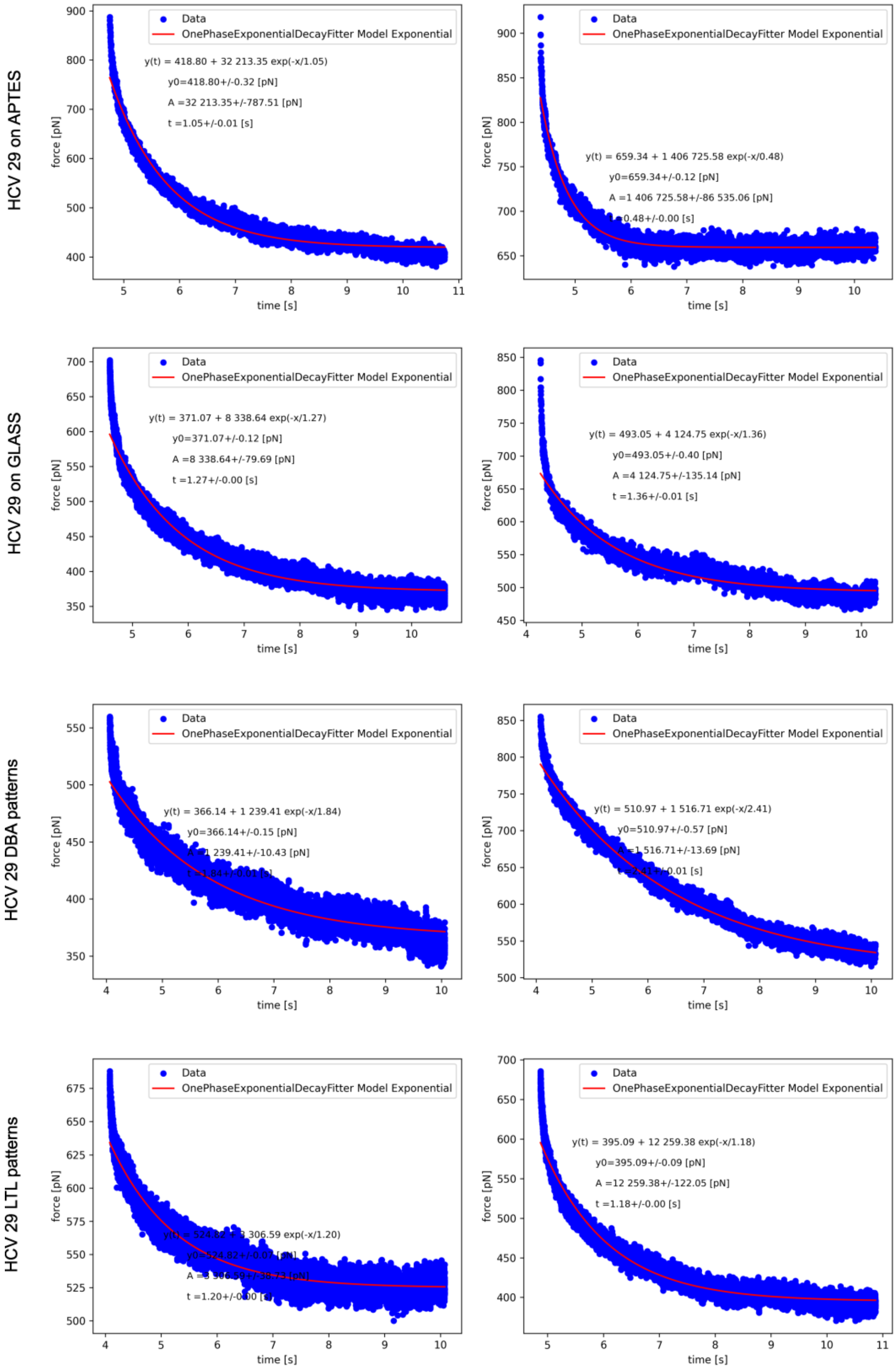

Figure S1. Examples of relaxation force curves recorded for HCV 29 cells grown on different types of substrates. Data points are in blue and fitted function in red. Parameter values corresponding to the fits are shown as insets in the plots.

Table S2. Number of cells measured to obtain parameters characterizing viscoelastic properties of HCV 29, 5637 and T24 cells cultured on glass and APTES-functionalized homogeneous substrates and lectin patterns.

# of cells (N)	glass	APTES	Lectin patterns (stripes)		
			LTL	*WGA*	*DBA*
HCV 29	54	66	62	-	36
T24	68	26	-	39	28
5637	44	41	72	-	71

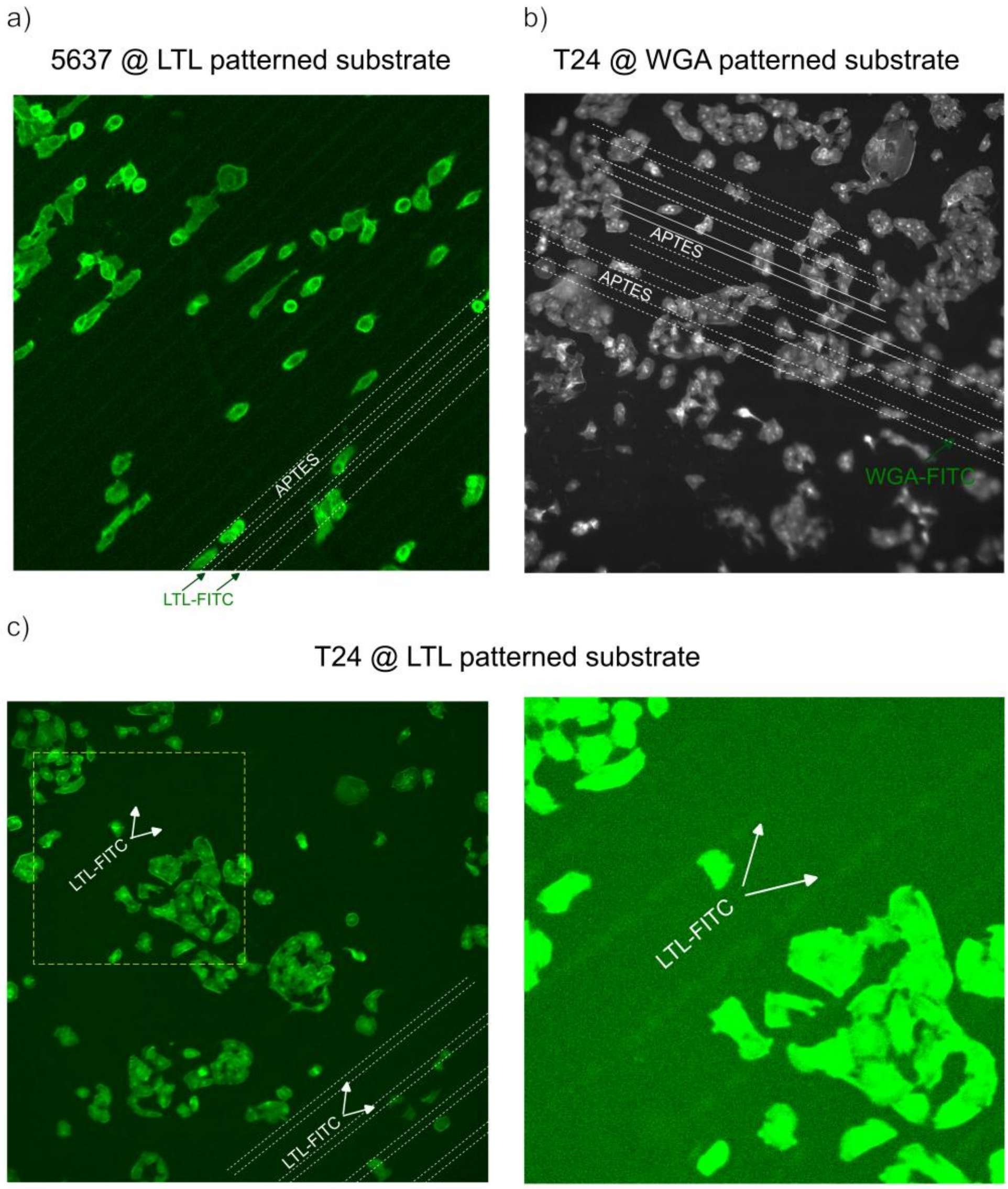

Figure S2. Fluorescence images showing the arrangement of 5637 (a) and T24 (b, c) cells on lectin-patterned substrates. (a) 5637 cells grow and proliferate on APTES between lectin stripes.

5637 cells exhibit higher sensitivity to the geometry of the biopattern. (b) T24 are mostly almost insensitive to the presence of the WGA stripes, and a small population of T24 cells changed their morphology due to the geometry of the lectin patterns. (c) T24 are insensitive to the presence of the LTL stripes. In the wright panel, zoomed area marked with white rectangle in the figure (left panel, c). The size of the image is 665.6 μm by 665.6 μm.

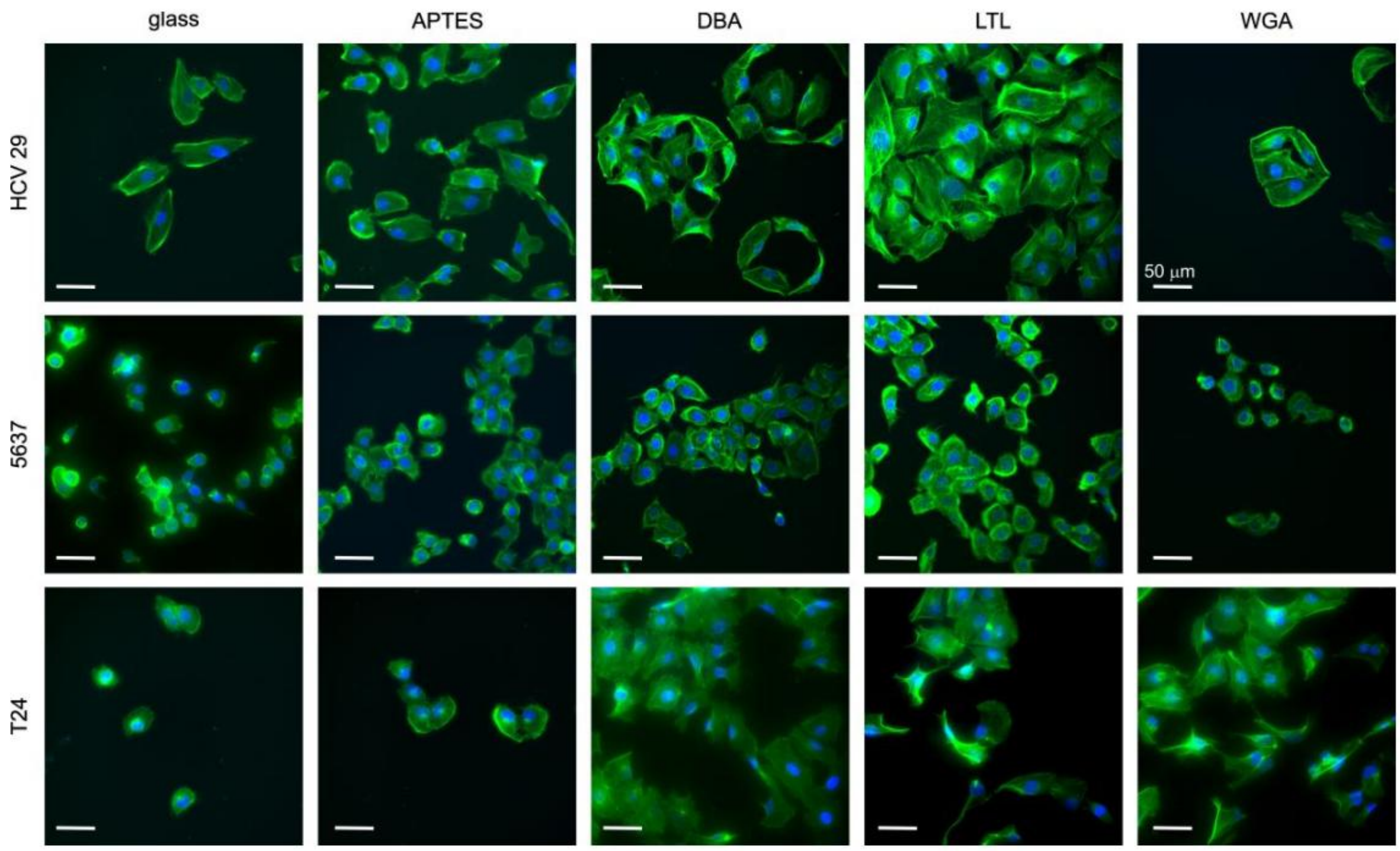

Figure S3. Actin filaments organization in HCV29, 5637, and T24 cells cultured on glass, APTES-functionalized glass and homogeneous DBA-, LTL- and WGA-coatings. Scale bar 50 μm.

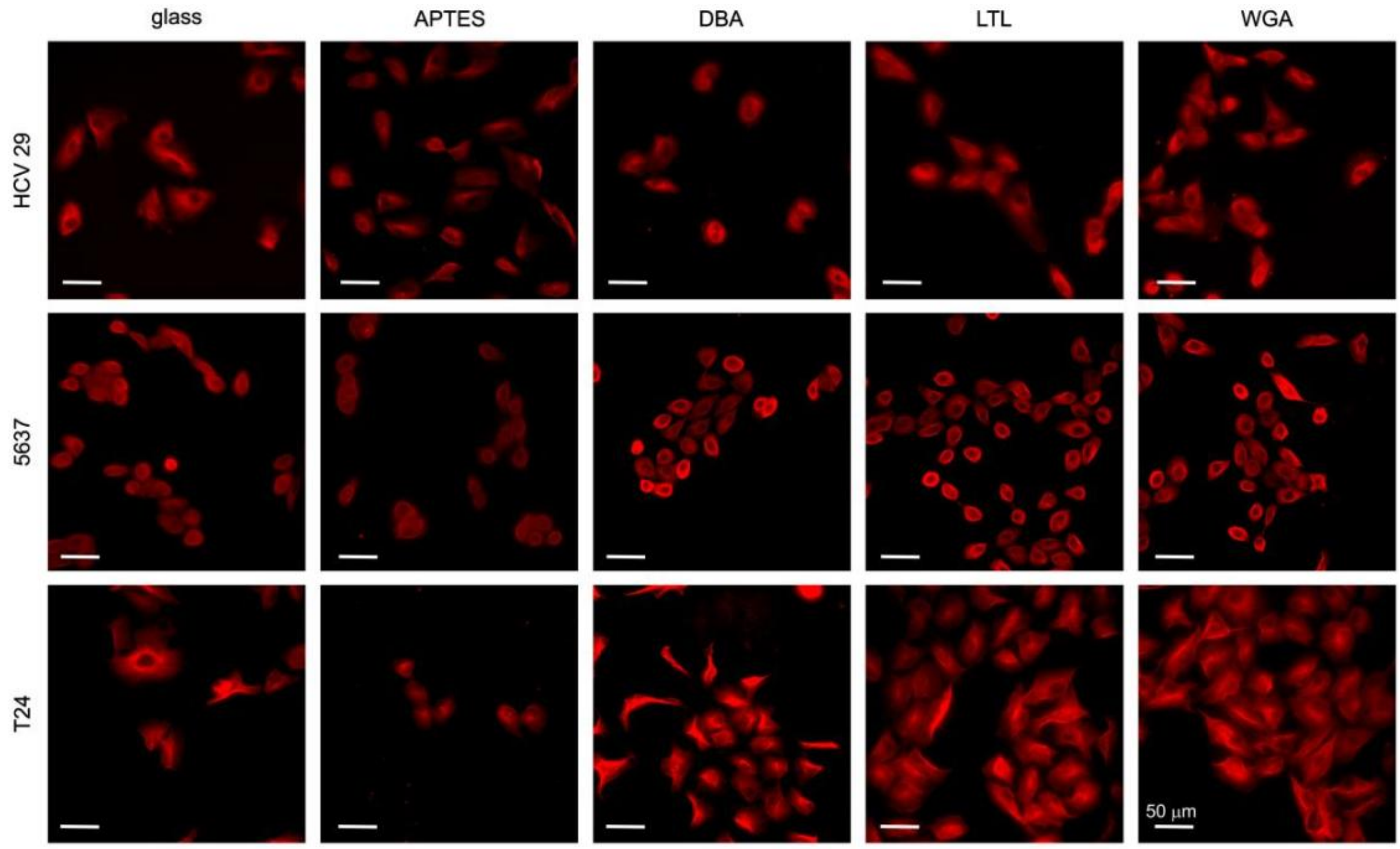

Figure S4. Microtubules organization in HCV29, 5637, and T24 cells cultured on glass, APTES-functionalized glass and homogeneous DBA-, LTL- and WGA-coatings. Scale bar 50 µm.

Fibronectin staining

Bladder cancer cells (HCV29, T24, and 5637) were seeded in 24-well black plates with clear bottoms (SensoPlate, Biokom, Janki, Poland) at a density of 1×10^4 cells per well in 0.5 mL of the appropriate culture medium. After 24 or 48 h of culture, the cells were washed three times with PBS and fixed with 3.7% paraformaldehyde (PFA) in PBS for 20 min at room temperature (RT). Following fixation, the cells were washed with PBS and permeabilized with cold 0.2% Triton X-100 in PBS for 5 min at 4°C. After three additional washes with PBS, nonspecific binding sites were blocked with 1% bovine serum albumin (BSA) in PBS for 1 h at RT.

Fibronectin was detected using mouse monoclonal anti-fibronectin antibodies (clone FN-15, F7387, Sigma-Aldrich, Poznań, Poland) at a concentration of 50 µg/mL in PBS. Cells were incubated with the primary antibody solution for 2.5 h at RT. Subsequently, the samples were

washed three times with PBS and incubated for 1 h at RT with Goat Anti-Mouse IgG (H+L) Cross-Adsorbed Secondary Antibody conjugated with Alexa Fluor 594 (Thermo Fisher Scientific, Waltham, MA, USA) at a concentration of 50 μg/mL in PBS. After secondary antibody incubation, the cells were washed five times with PBS. Cell nuclei were stained with Hoechst 34580 diluted 1:5000 in PBS. Following nuclear staining, the samples were washed five times with PBS and prepared for imaging.

Fluorescence microscopy

Fluorescence images were acquired using an inverted fluorescence microscope (Olympus IX83) equipped with a Photometrics Prime BSI Express camera (2048 × 2048 px), a 100 W mercury lamp, and appropriate fluorescence filters. Images were collected using a 40× objective, corresponding to a field of view of 332.8 μm × 332.8 μm. Exposure times were set to 30 ms for fibronectin (Texas Red channel) and 10 ms for nuclei (blue channel). All images were acquired using identical microscope settings and exposure times to enable direct comparison of fluorescence intensity between samples. Images were acquired using CellSens software (Olympus) and processed using ImageJ (version 1.54p).

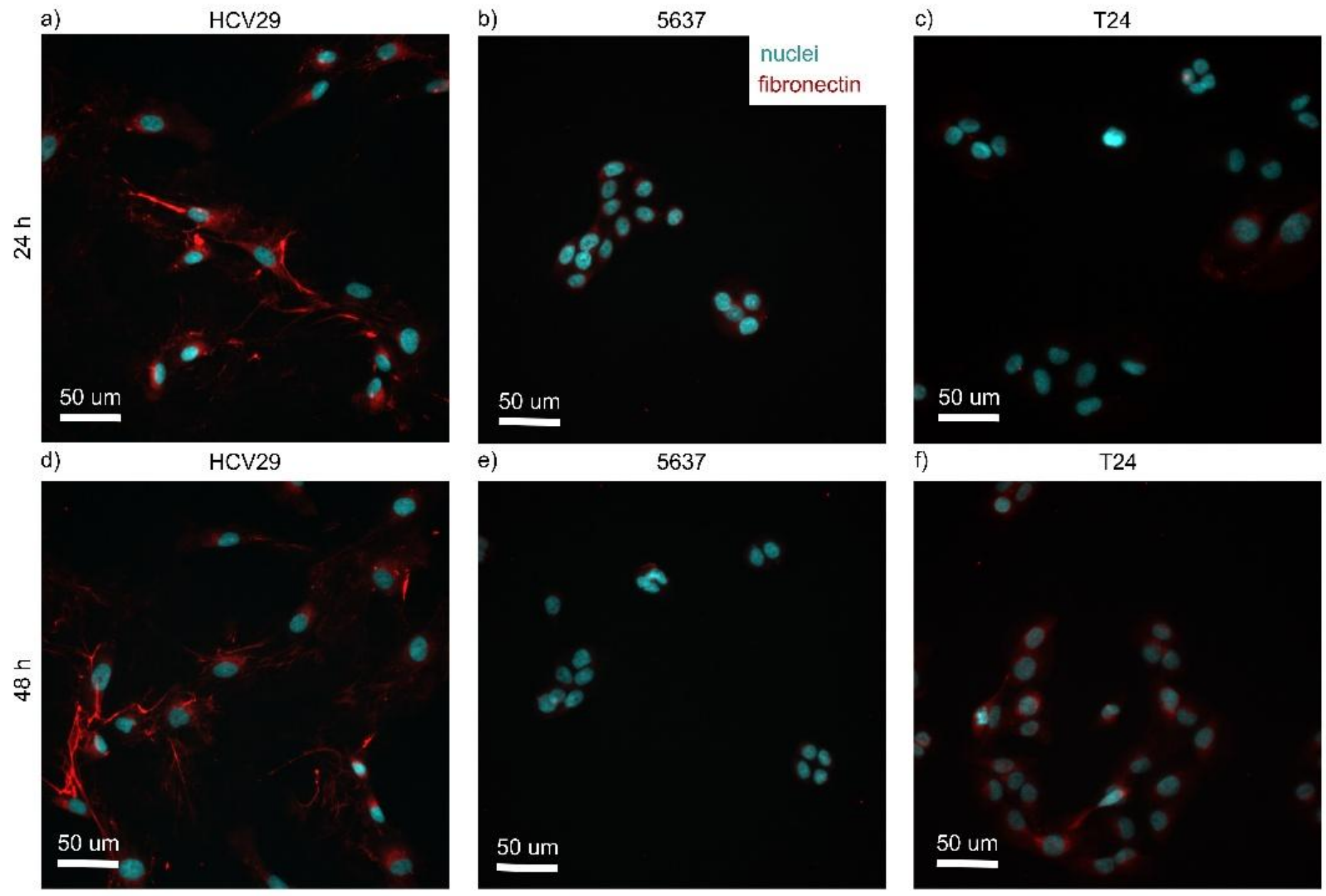

Figure S5. (a) Fluorescence image of fibronectin (Fn) secreted by HCV29 (a, d), 5637 (b, e), and T24 (c, f) cells after 24 (a-c) and 48 (d-f) hours of culture on glass coverslips. Fn is red and nuclei are cyan.

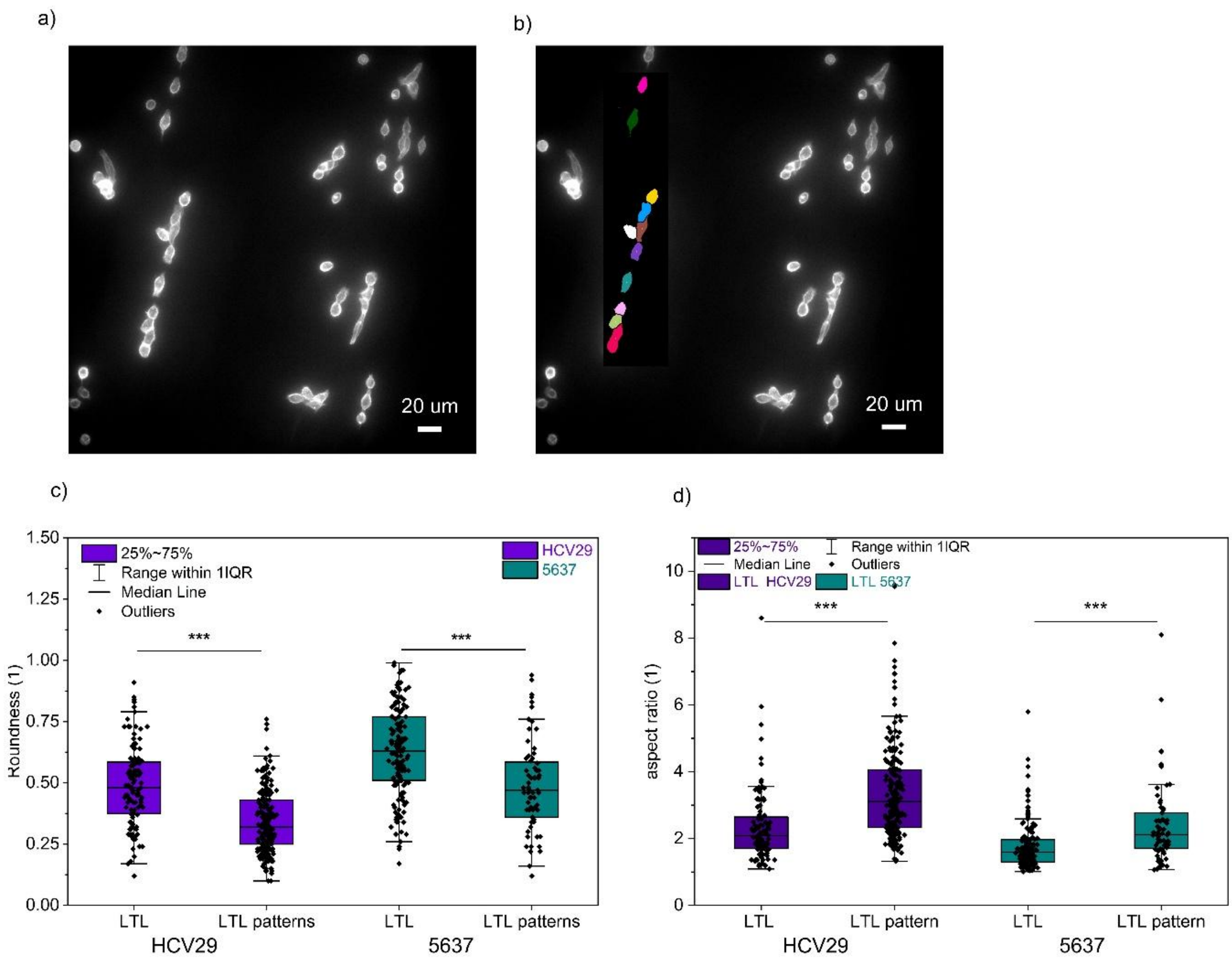

Figure S6. Quantitative analysis of cell morphology for HCV29 and 5637 cells cultured on homogeneous LTL-coated substrates and LTL micropatterns (three biological replicates). (a) Representative 40× fluorescence image of 5637 cells cultured on an LTL-coated substrate. (b) Overlay of the ImageJ-generated mask used for particle segmentation and shape analysis. Box plots show aspect ratio and roundness for HCV29 and 5637 cells cultured on homogeneous LTL coatings and LTL patterns. Cells cultured on LTL patterns exhibited higher aspect ratio and lower roundness, indicating increased cell elongation compared with cells cultured on homogeneous LTL coatings.

Table S3. The apparent Young's modulus (*E*) obtained for HCV 29, 5637, and T24 cells cultured on homogeneous glass and APTES-functionalized substrates, and lectin patterns (LTL, DBA and WGA stripes). Data presented as medians ± MAD.

E (Pa)	*HCV 29*	*T24*	*5637*
glass	1078 ± 434	1016 ± 595	664 ± 234
APTES	832 ± 265	1024 ± 307	440 ± 131

striped DBA patterns	879 ± 315	1133 ± 479	642 ± 215
striped LTL patterns	1260 ± 520	-	566 ± 220
striped WGA patterns	-	1725 ± 598	-

Table S4. The equilibrium elastic modulus (E_{inf}) obtained for HCV 29, 5637, and T24 cultured on homogeneous glass and APTES-functionalized substrates, and lectin patterns (LTL, DBA and WGA stripes). Data presented as median ± MAD.

E_{inf} *(Pa)*	*HCV 29*	*T24*	*5637*
glass	641 ± 68	632 ± 69	407 ± 53
APTES	253 ± 73	705 ± 114	272 ± 22
striped DBA patterns	685 ± 104	1077 ± 251	395 ± 47
striped LTL patterns	519 ± 70	-	351 ± 31
striped WGA patterns	-	1000 ± 78	-

Table S5. Deborah numbers (*De*) obtained for HCV 29, 5637, and T24 cultured on homogeneous glass and APTES-functionalized substrates, and lectin patterns (LTL, DBA and WGA stripes). Data presented as median ± MAD.

De	*HCV 29*	*T24*	*5637*
glass	0.292 ± 0.053	0.259 ± 0.031	0.249 ± 0.026
APTES	0.270 ± 0.039	0.293 ± 0.036	0.264 ± 0.022
striped DBA patterns	0.344 ± 0.046	0.292 ± 0.053	0.302 ± 0.042
striped LTL patterns	0.308 ± 0.043	-	0.289 ± 0.039
striped WGA patterns	-	0.325 ± 0.049	-

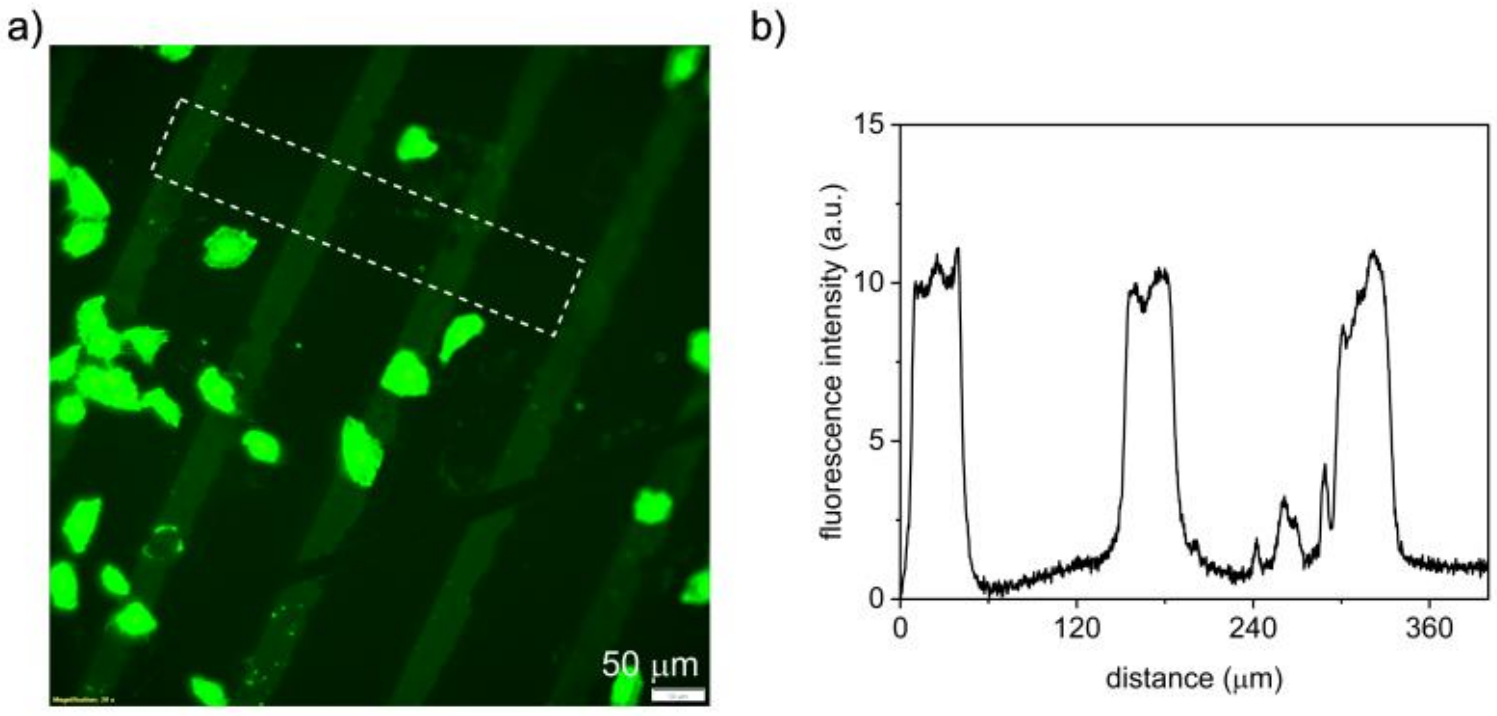

Figure S7. (a) Fluorescence image of DBA patterns with HCV 29 cells (light green). (b) Fluorescence intensity along the DBA pattern obtained from the area in (a) indicated by white dashed lines.
